# FedscGen: privacy-preserving federated batch effect correction of single-cell RNA sequencing data

**DOI:** 10.1186/s13059-025-03684-6

**Published:** 2025-07-22

**Authors:** Mohammad Bakhtiari, Stefan Bonn, Fabian Theis, Olga Zolotareva, Jan Baumbach

**Affiliations:** 1 Institute for Computational Systems Biology, University of Hamburg, Hamburg, Germany; 2 Institute of Medical Systems Bioinformatics, Center for Biomedical AI (bAIome), Center for Molecular Neurobiology (ZMNH), University Medical Center Hamburg-Eppendorf, Hamburg, 20246 Germany; 3 German Center for Child and Adolescent Health (DZKJ), Partner Site Hamburg, University Medical Center Hamburg-Eppendorf, Hamburg, 20246 Germany; 4 Hamburg Center for Kidney Health (HCKH), University Medical Center Hamburg-Eppendorf, Hamburg, 20246 Germany; 5 Hamburg Center for Translational Immunology (HCTI), University Medical Center Hamburg-Eppendorf, Hamburg, 20246 Germany; 6 School of Life Sciences Weihenstephan, Technical University of Munich, Munich, Germany; 7 Helmholtz Zentrum München – German Research Center for Environmental Health, Institute of Computational Biology, Neuherberg, Germany; 8 Department of Mathematics, Technical University of Munich, Munich, Germany; 9 Data Science in Systems Biology, TUM School of Life Sciences, Technical University of Munich, Freising, Germany; 10 Department of Mathematics and Computer Science, University of Southern Denmark, Odense, Denmark

**Keywords:** Batch effect correction, Federated learning, Privacy-preserving computation, Single-cell RNA-seq, Secure multiparty computation (SMPC), Generative models

## Abstract

Single-cell RNA-seq data from clinical samples often suffer from batch effects, but data sharing is limited due to genomic privacy concerns. We present FedscGen, a privacy-preserving communication-efficient federated method built upon the scGen model, enhanced with secure multiparty computation. FedscGen supports federated training and batch effect correction workflows, including the integration of new studies. We benchmark FedscGen across diverse datasets, showing competitive performance—matching scGen on key metrics like NMI, GC, ILF1, ASW_C, kBET, and EBM on the Human Pancreas dataset. Published as a FeatureCloud app, FedscGen enables secure, real-world collaboration for scRNA-seq batch effect correction.

**Supplementary Information:**

The online version contains supplementary material available at 10.1186/s13059-025-03684-6.

## Background

Over the years, advancements in technology have significantly enhanced our ability to generate single-cell gene expression data. This data, often derived from a range of experiments, introduces variations because of differences in capture times, personnel, equipment, and technological platforms. These variations result in batch effects, which can overshadow the actual biological differences of interest. Addressing these discrepancies is essential, and tools like ComBat [1] and limma’s removeBatchEffect [2, 3], originally developed for batch effect correction in microarray data, have been utilized for bulk RNA [4] and applied to simulated single-cell RNA sequencing (scRNA-seq) data [5]. However, single-cell data is characterized by a high prevalence of dropouts that emerge due to the stochasticity of gene expression [6] and fragment sampling. This necessitated the development of specialized methods to cater to such data intricacies [7–9]. Concurrently, the capability to profile thousands of cells in one experiment has given rise to significant projects like the Human Cell Atlas [10] which deepens our understanding of cell types and states. Gaining comprehensive insight, especially encompassing both healthy and diseased cells, requires merging datasets from different sources and platforms [11, 12].

The necessity of addressing batch effects in scRNA-seq data has led to various computational strategies. First, there are adaptations of tools originally designed for bulk transcriptome data, such as ComBat (1) and limma (2), which have been modified to suit sequencing data [13]. Second, a significant subset of methods employs mutual nearest neighbors (MNNs) and its following improvements such as fastMNN to align data [8]. Other techniques like Scanorama [11] and BBKNN [12] also employ MNNs in reduced spaces. In addition, Harmony [14] offers a robust approach by utilizing PCA for dimensionality reduction and an iterative process to refine cell clustering across batches. The Seurat package introduced MultiCCA [7], later evolving to Seurat 3 [15] using MNNs within a canonical correlation analysis.

Neural network-based approaches gain an edge over traditional methods [16]. In fact, even with relatively simple architectures, the adaptability and learning capabilities of neural networks offer substantial benefits for handling and interpreting the growing volumes of data characteristic of modern biological and genomic studies. Accordingly, recent advancements in deep learning have led to novel approaches such as residual neural network strategy [17] and scGen [18], a Variational AutoEncoder (VAE) model. Recently, approaches like DISCERN [19] have applied deep neural networks (DNNs) to scRNA-seq datasets with high-quality references to improve data integration and biological coherence across conditions. scGen [18] utilizes the architecture of a VAE to generate new data points while maximizing the likelihood of each sample using a generative process based on latent variables that are sampled to compute the probability of observing a data point given the model parameters.

With the growing demand for large-scale single-cell datasets to enable robust and generalizable analyses, a central challenge arises: balancing the necessity of data aggregation with the need to protect individual privacy. While combining scRNA-seq datasets across studies and institutions enhances analytical power, it also introduces significant legal and ethical concerns, particularly under data protection regulations such as the GDPR (Article 4(1)) [20]. Although single-cell data may appear anonymized, it can still reveal highly personal biological traits unique to individuals or their relatives. This creates risks not only of re-identification but also of unintended disclosure of sensitive information. Studies have shown that despite inherent variability in single-cell measurements, genotype–phenotype associations derived from scRNA-seq data, in specific expression count matrices [21], can be used to infer private attributes [22], and that combining multiple datasets may even allow adversaries to reconstruct identifiers such as surnames [23] or addresses [24]. Furthermore, single-cell gene expression datasets are vulnerable to linking attacks, where adversaries infer sensitive phenotypic information using publicly available tissue or cell-type-specific expression quantitative trait loci (eQTL) data [21].

Federated learning constitutes a solution, allowing machine learning without central data aggregation, addressing privacy and legal challenges [25–27]. Federated learning (FL) [28] presents a paradigm shift in machine learning by allowing for the training of algorithms across decentralized datasets without the need to share the raw data itself, thereby preserving privacy and ensuring data security. FL approaches can cope with heterogeneous data sources [29, 30] and imbalanced data across clients [31]. Federated platforms like FeatureCloud [32] were designed for facilitating development and deployment of federated applications in the field of biomedicine. Previously, federated analyses were conducted, either without batch effect corrections [33] or by explicit accounting for batch effects in the model [34].

In this paper, leveraging scGen’s strengths in handling diverse datasets [35], we introduce FedscGen, a federated framework for privacy-aware batch effect correction of distributed scRNA-seq data. We deploy the VAE model from scGen across multiple clients where each client trains the model on its own dataset. Afterwards, clients share the trained parameters with a coordinator which aggregates and updates the global model by averaging the parameters, and broadcasts the global model back to all clients. To enable batch correction without data sharing, we also introduce the Federated $\delta$ -vector estimation and correction procedure that identifies dominant batches for shared cell types and enables local correction based on securely aggregated latent representations. FedscGen supports the entire process in a privacy-aware manner using secure multiparty computation (SMPC) based on an additive secret sharing technique [36, 37]. We demonstrate that FedscGen achieves comparable performance to scGen on benchmark datasets while addressing the critical privacy constraints of multi-center studies.

## Results

We introduce FedscGen, a novel federated learning framework for enabling collaborative batch effect correction based on the scGen model for training local VAE models in a privacy-aware federated manner. FedscGen employs two federated workflows (WFs): training (for training the VAE model) and correction (for calculating mean latent features for shared cell types to enable batch effect correction).

To evaluate FedscGen, we applied it to eight benchmark datasets and simulated heterogeneous scenarios by assigning each of the batches in a dataset to separate clients. This setup reflects real-world differences in data distribution due to varying geographic and procedural collection protocols. In certain experiments, we excluded one batch from the training workflow and designated it as a held-out batch, which was only included in the correction workflow. This configuration enabled the evaluation of FedscGen’s ability to perform batch effect correction during the integration of new studies. In our experiments, we trained scGen for 100 epochs with 0.001 as the learning rate, a configuration that consistently removed batch effects across datasets [18]. Also, we incorporated an early stopping mechanism terminating training if the validation loss did not improve beyond a specified threshold for a predetermined number of epochs [38].

We benchmarked FedscGen against scGen for batch effect correction using both visual and quantitative assessments. We assessed Uniform Manifold Approximation and Projection (UMAP) plots for batch effect correction, and additionally measured the quality of batch mixing and integration using kBET (*k*-nearest neighbor batch-effect test) acceptance rate and KNN-accuracy, which assess how well samples from different batches mix after correction, indicating the effectiveness of batch integration. Additionally, we used ASW (Average Silhouette Width) and NMI (Normalized Mutual Information) to evaluate the cohesion and separation of clusters from different batches, indicating how well the correction method maintains cell type distinctions while integrating data. Moreover, we used EBM (Empirical Batch Mixing) to assess the empirical quality of batch correction. In another category of benchmarking metrics, we assessed biological signal preservation using LISI (Local Inverse Simpson’s Index), ILF1 (Inverse Local *F*1 Score), and GC (Graph Connectivity).

To compare performance across datasets, we benchmarked the quality of batch effect correction on the Cell Line (CL) [39], Human Dendritic Cells (HDC) [40], Human Pancreas (HP) [39, 41–45], Mouse Brain (MB) [46, 47], Mouse Cell Atlas (MCA) [48, 49], Mouse Hematopoietic Stem and Progenitor Cells (MHSPC) [50, 51], Mouse Retina (MR) [52, 53]**,** and human Peripheral Blood Mononuclear Cell (PBMC) [39] datasets. Throughout our experiments, we compared the performance of FedscGen and scGen by calculating the performance difference for each metric $m$ as $\triangle{}_m={\mathrm{FedscGen}}_m-{\mathrm{scGen}}_m$, resulting in $\triangle_m\in\left[-1,1\right]$, where positive values indicate better performance of FedscGen, and negative values indicate better performance of scGen (see the “ Methods” section). In a similar fashion, we also calculated the performance difference of FedscGen-SMPC relative to scGen.

### FedscGen enables privacy-aware batch effect correction of distributed data

**Fig. 1:**
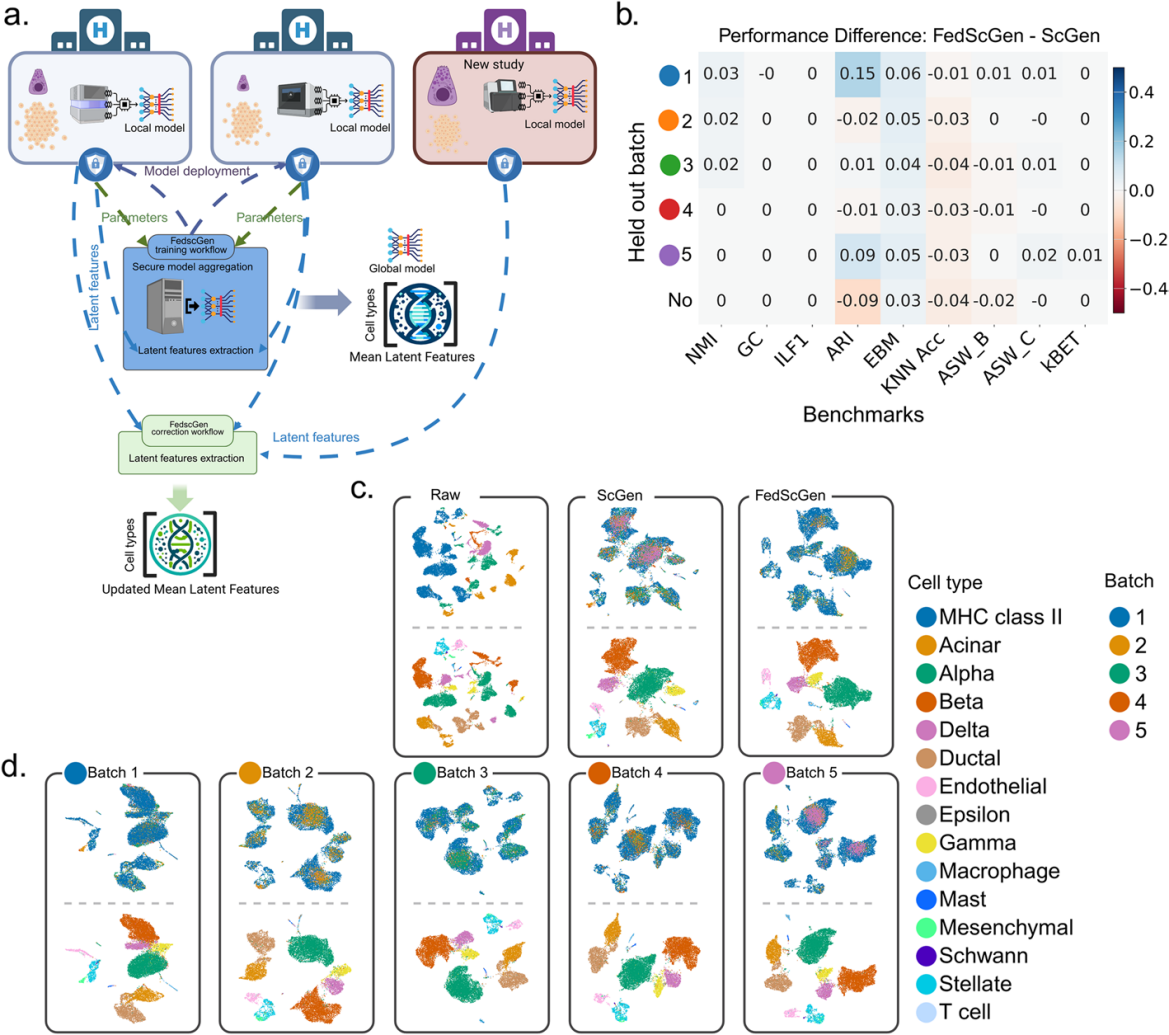
FedscGen delivers results comparable to scGen without compromising patient privacy. **a** FedscGen orchestrates federated training by deploying models to clients for local updates, followed by secure aggregation over communication rounds. The correction workflow allows FedscGen to correct batch effects and support the inclusion of new studies. **b** FedscGen achieves comparable batch effect correction to scGen across batch mixing and biological preservation metrics for different held-out batches in the Human Pancreas (HP) dataset. In the held-out scenario, the training WF includes four clients (each with a batch), and the correction WF incorporates the fifth batch as a new study for correction. In the no held-out scenario, all five batches are included as clients in both the training and correction workflows. **c** UMAP visualization of batch effect correction using FedscGen in a federated scenario with five clients, showing effective batch mixing and cell type separation. **d** UMAP plots for each held-out batch scenario demonstrating that FedscGen achieves correction quality comparable to centralized scGen without a held-out batch

In a real-world scenario, heterogeneous, identifiable scRNA-seq data resides in multiple institutions. Collecting data in a centralized custody would significantly benefit the analysis, but privacy concerns strongly discourage hospitals from sharing their data. FedscGen enables federated computation where a centralized coordinator orchestrates clients, e.g., hospitals and clinics, that collaboratively train models using patients’ distributed data but without compromising data privacy (Fig. 1a). In the FedscGen training workflow, the coordinator deploys a VAE model, with common initial parameters shared with all clients. Each participant trains the model locally for the $e$ epochs, then sends the trained parameters to the coordinator. The coordinator securely aggregates the parameters of local models and outputs the global model parameters (Fig. 1a). To aggregate the models, we use FedAvg [28] with factoring the ratio of training samples for client c in averaging the parameters $\theta_r\leftarrow{\textstyle\sum_{c\in {\mathcal{C}}}}N_c\cdot\theta_c$, where $\theta_r$ is the global weights in *r*th communication round and $\mathcal{C}$ is the number of clients. After training the model, the latent representations of local cells are corrected by subtracting each latent vector from mean latent features of the dominant batch (see the “ Methods” section).

**Fig. 2:**
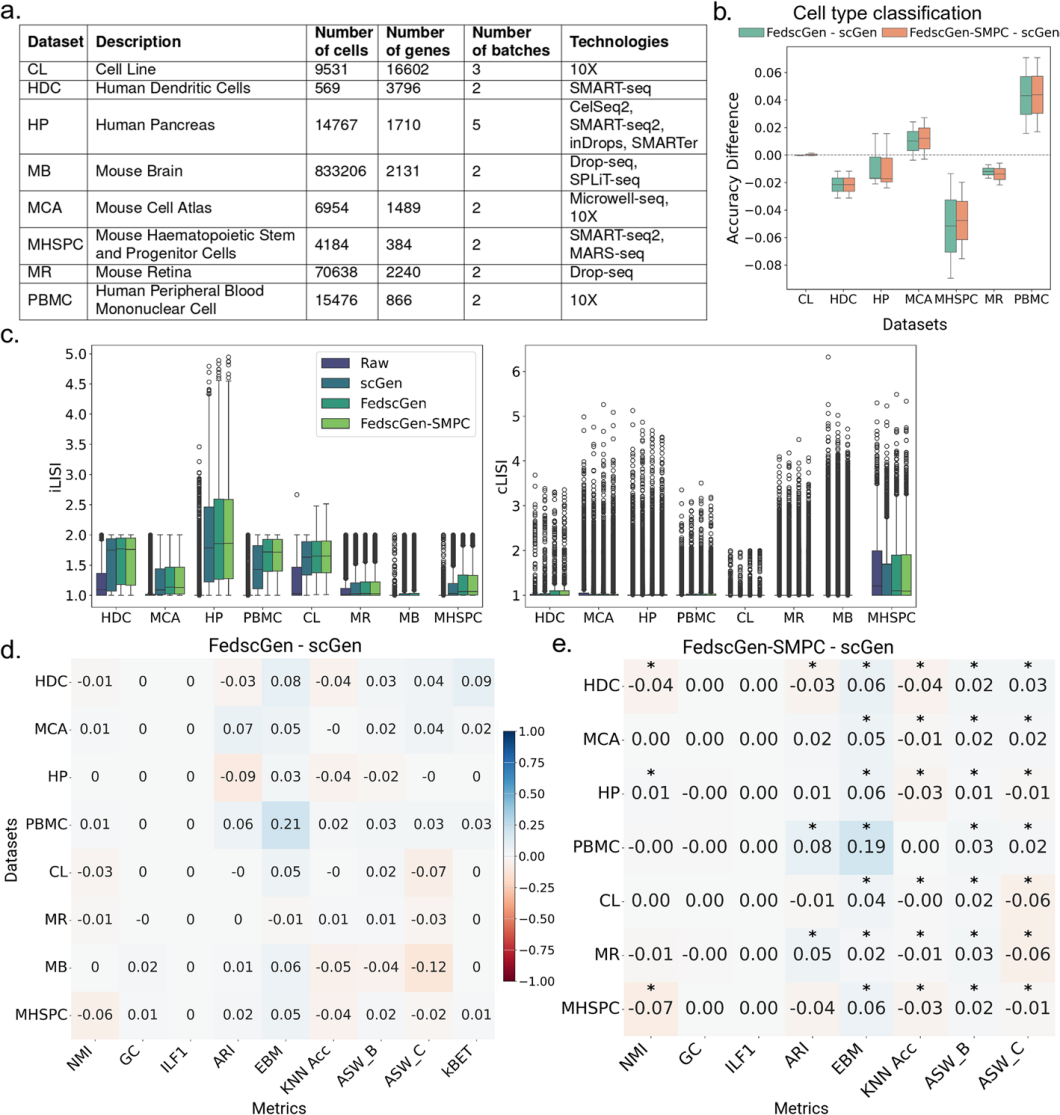
The results of FedscGen benchmarking (with and without SMPC).** a** Summary of datasets used for benchmarking.** b** Comparison of classification accuracy for cell type prediction using corrected HP data from FedscGen, FedscGen-SMPC, and scGen. **c** Comparing iLISI and cLISI scores, left and right, respectively, across eight datasets, Both scGen and FedscGen perform comparably well. **d** Performance difference between scGen and FedscGen on various benchmarking metrics. **e** FedscGen with SMPC yields comparable results with no significant difference, based on the Mann Whitney test, for GC and ILF1, and better results for EBM and ASW_B across seven datasets. While a performance difference $\triangle{{}}_m$ value shows which method performs better, the cells are marked with asterisks if the observed performance difference is statistically significant

In a FedscGen workflow with five clients, where each was assigned one of the five batches in Human Pancreas (HP) dataset [39, 41–45] (Fig. 1b–c), we trained FedscGen for eight communication rounds and two local epochs. By comparing mean kBET acceptance rates, we showed both methods are equally well in batch mixing while making cell types more distinguishable in the HP dataset (Fig. 1b–c), evidenced by similar performance in the NMI and ASW_C metrics. Also, the identical ILF1 and GC scores indicate comparable preservation of biological signals and maintenance of biological connectivity. Furthermore, FedscGen outperforms scGen by 0.03 in EBM, indicating better batch mixing, it lags behind scGen in ARI by 0.09, KNN Acc by 0.04, and ASW_B by 0.02 (Fig. 1b, no held-out batch). FedscGen shows decent batch effect removal with the inclusion of new studies (Fig. 1d), where batches are sequenced by different technologies—CelSeq2, SMART-seq2, inDrops, and SMARTer [39, 41–45] (Fig. 2a)—one of which is used as a held-out batch. Furthermore, evaluation of the effectiveness of correction on a downstream cell type classification task using corrected data from FedscGen versus scGen, yields similar results, where FedscGen's performance difference stays consistently less than 2% across various metrics when tested on different batches of HP dataset (Fig. 2b). In terms of iLISI scores, the variation of batches in raw HP dataset is not wide and both FedscGen and scGen notably increase the variation while FedscGen provides a wider distribution (Fig. 2c). In terms of cLISI, the diversity of batches in the raw HP dataset is very low, and both scGen and FedscGen manage to preserve the low diversity of cell types within each cell neighborhood after correcting the data (Fig. 2c).

### FedscGen corrects batch effects of new studies without compromising patients’ data

In FedscGen’s correction WF with inclusion of new studies that were absent in the training WF, the mean latent features of the dominant batch for some cell types could be updated. In a training workflow with four clients, each representing one unique batch of the HP dataset, we trained a VAE model in a federated fashion. Using the global model, in the correction workflow with five clients, we corrected all five batches in the HP dataset, including the held-out batch (Fig. 1b, d). We also trained the scGen model and corrected the HP data under the same setting (Fig. 1b). In all five held-out scenarios, FedscGen performs comparably well in comparison to scGen; for KNN_Acc with at most 0.04, scGen performs better, and for some of the held-out batches, it shows minor improvement of at most 0.02 in ASW_B and ARI. In comparison with batch effect correction of data without any held-out studies in the training (Figs. 1b–d and 2d (for no held-out batch)), FedscGen manages to preserve the distinguishability of cell types while mixing the batches for various held-out batches well.

### FedscGen-SMPC corrects batch effects by securely aggregating the models without revealing the local model parameters

Comparing the performance of FedscGen-SMPC and scGen across seven datasets and eight metrics, we categorically evaluated the batch integration and biological preservation of both methods (Fig. 2e). We trained FedscGen-SMPC for eight communication rounds and two local epochs. Using the Wilcoxon-Mann–Whitney test across 10 random seeds per method, followed by Benjamini–Hochberg correction, we assessed whether the observed differences were statistically significant (adjusted *p* < 0.05). Statistically significant differences for any metric-dataset pair are marked with asterisks in Fig. 2e. In terms of consistent significant differences for the same metric across all datasets, there were no differences in GC and ILF1, while FedscGen significantly outperformed scGen in EBM and ASW_B. For the remaining metrics, there were no consistent significant differences in favor of one method across all datasets. For instance, scGen significantly outperformed FedscGen in biological signal preservation metrics such as ASW_C and ARI in the CL and MHSPC datasets. Additionally, scGen showed significantly better batch mixing in terms of NMI in the HDC and MHSPC datasets. These results partially demonstrate a trade-off between batch effect removal and biological preservation, with FedscGen achieving competitive or superior integration in many cases while exhibiting a controlled and interpretable impact on biological fidelity. Meanwhile, in terms of the effect of batch effect correction on the downstream cell type classification task, FedscGen-SMPC consistently performs comparably to FedscGen, with only minor differences in mean and variance across all datasets (Fig. 2b). Similarly, FedscGen-SMPC yields iLISI and cLISI scores (Fig. 2c) that closely match those of FedscGen, demonstrating robust performance while enhancing privacy across all datasets.

### Comprehensive comparison of FedscGen against scGen on eight datasets using ten evaluation metrics

We use UMAP as a visualization technique, batch effect correction metrics, and quality assessment of downstream analysis based on cell type classification accuracy of a MLP model trained on raw vs corrected data. A summary of dataset characteristics is provided in Fig. 2a (for more details see Additional file 1: Tables S1–2). All datasets, except for HP and CL, with 5 and 3 batches, respectively, are constructed with exactly two batches. In all scenarios, we use the same VAE architecture and generic default hyperparameters.

FedscGen achieves better results or performs as good as scGen across various datasets, while only showing negligible differences in a few metrics (Fig. 2d). FedscGen consistently performs better than or equal to scGen for the MCA and PBMC datasets across all metrics, and for the GC, ILF1, and kBET metrics across all datasets, indicating robust preservation of biological signals while effectively removing batch effects. Also, in terms of iLISI, both models show notable improvement of batch mixing, in comparison to the raw data, across all datasets by increasing the diversity of batches in the neighborhood of various cells (Fig. 2c). However, scGen occasionally shows marginal advantages in certain metrics; in four cases, for NMI, ARI, and or ASW_C, we observe modest outperformance of scGen by a margin of 0.06 to 0.12, indicating better cell type distinguishability (Fig. 2d). We demonstrate later how using different numbers of local epochs and communication rounds can make FedscGen's results more competitive (Additional file 2: Fig. S1).

**Fig. 3:**
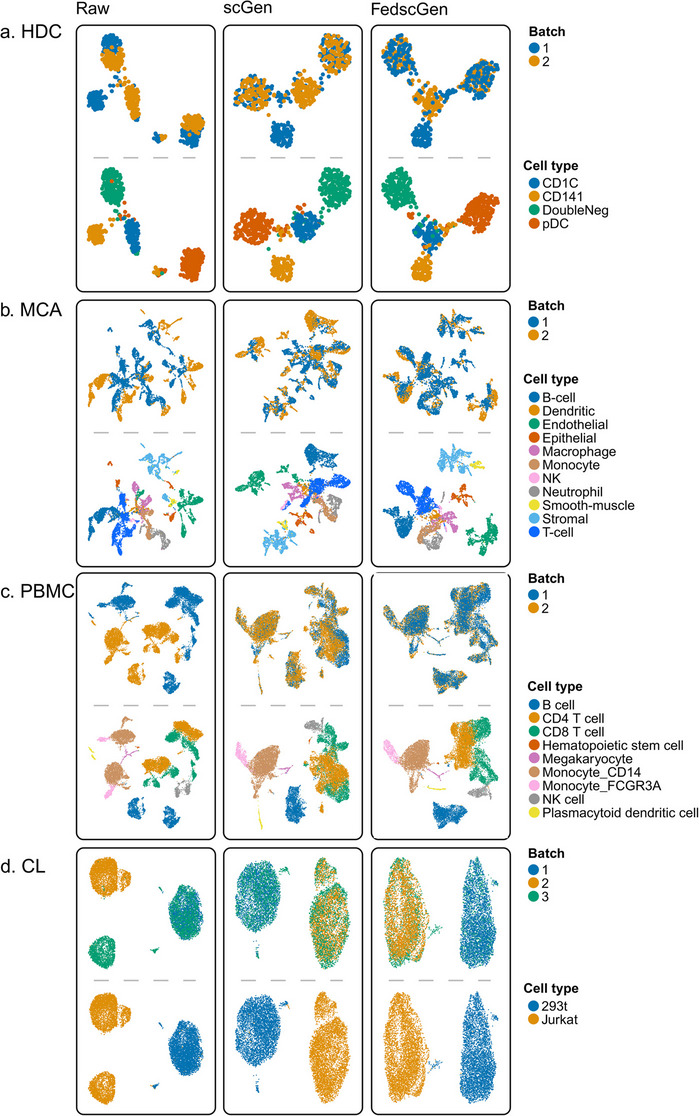
FedscGen removes batch effects by mixing batches and separating cell types in a manner comparable to scGen. UMAPs of cells colored by cell types and batches are shown for raw and corrected data using scGen and FedscGen: **a** HDC, **b** MCA, **c** PBMC, and **d** CL datasets

In the HDC dataset, which is relatively small with 576 cells sequenced using SMART-seq technology, we observed that the cell types were fairly separated by batch in the raw data, while the raw data shows the high batch diversity (Fig. 2c). Both scGen and FedscGen have effectively mitigated the batch effect, which is crucial for accurate analysis within such a specialized cell population. FedscGen shows a notable advantage in reducing batch effects, as indicated by kBET, ASW_B, and EBM metrics (Fig. 2d). Both methods preserve biological signals, according to GC and ILF1 metrics. However, the UMAP visualization (Fig. 3a) shows that scGen has slightly better clusters of standalone cell types, those present only in one batch, CD141 and CD1C, resulting in a 0.03 improvement on ARI (Fig. 2d). Nonetheless, by training FedscGen for 6 rounds and 2 epochs, FedscGen achieves ARI differences of 0 (Additional file 2: Fig. S1).

The MCA dataset, which suffers from batch effects due to different sequencing methodologies (microwell-seq and 10X technologies), exhibits low diversity in batches as indicated by iLISI (Fig. 2c). However, it has shown improvement in the integration of cells across batches with the application of both scGen and FedscGen. Particularly noteworthy is the distribution of cell types such as B-cells, T-cells, and endothelial cells; these initially displayed a skewed batch distribution but appear more homogeneously distributed after correction, as visualized in Fig. 3b. Across the metrics, FedscGen demonstrates consistently better or comparable results to scGen.

In the PBMC dataset, which shows low iLISI diversity among batches (Fig. 2c), cell types such as B cells, Monocyte CD14, and CD4T cells are clearly separated across batches (Fig. 3c) in raw data where both models manage to cohesively cluster the cell types while mixing the batches. FedscGen consistently performs as well as or better than scGen across all metrics, with a notable edge of 0.21 in EBM (Fig. 2d).

In the CL dataset, which comprises 2,885 cells from 293 T and 3,258 from Jurkat, the raw data's iLISI suggests batch diversity (Fig. 2c). This diversity is evident in the distribution of the 293 T cell type across batches, while the Jurkat cell type clearly suffers from a batch effect (Fig. 3d). Both approaches perform equally well across all metrics with a margin of 0.03 difference, except for ASW_C, where scGen has a modest edge of 0.07 (Fig. 2d).

For the MR dataset, which includes a large number of cells (26,830 from Drop-seq and 44,808 from another technology), the raw data exhibits modest diversity in batches (Fig. 2c). This diversity is attributed to the distribution of rods and Müller cell types across batches, while Bipolar cell type clearly suffers from batch effect (Additional file 2: Fig. S2a). Both methods perform well across all metrics, except for ASW_C, where scGen performs slightly better by 0.03 (Fig. 2d).

The MB dataset comprises 691,600 cells from Drop-seq and 141,606 cells from SPLiT-seq, resulting in considerable imbalance and low diversity across batches and cell types (Fig. 2c). It exhibits significant batch separation among Neuron and Olfactory cell types (Additional file 2: Fig. S2b). Both FedscGen and scGen yield competitive performance across several metrics (Fig. 2d). FedscGen achieves an improvement of 0.06 in EBM (Fig. 2d), demonstrating the empirical effectiveness of its batch correction. On the other hand, scGen’s 0.12 advantage in ASW_C (Fig. 2d) signifies its strength in maintaining cohesiveness of cell types.

The MHSPC dataset, comprising a moderate number of cells (1920 from SMART-seq2 and 2729 from MARS-seq), with low diversity in batches shows the highest diversity in cell types among the datasets (Fig. 2c), where CMP and MEP cell types are distributed among the batches (Additional file 2: Fig. S2c). FedscGen performs better or comparably across most metrics. However, scGen shows modestly better performance in NMI and KNN_ACC, by 0.06 and 0.04, respectively, indicating better cell type distinguishability and batch integration (Fig. 2d).

### Tuning of the number of communication rounds and local training epochs improves the results of FedscGen compared to scGen

For the hyperparameter tuning of FedscGen across datasets, we evaluated the model using NMI, ARI, ASW_B, ASW_C, KNN_Acc, and EBM metrics (Additional file 2: Fig. S1). Training the FedscGen model for additional communication rounds with the same number of epochs does not necessarily enhance performance. A similar trend is noted when increasing the number of local epochs. Accordingly, we cannot define a one-size-fits-all guideline for all datasets, and achieving optimal performance may require trade-offs across metrics. Given the importance of communication efficiency in federated solutions, reducing the number of rounds while minimizing performance loss is preferred. With this consideration, and to maintain simplicity through generic values for all settings, we find that the best performance across all datasets and metrics can be achieved with 2 epochs and 8 rounds. In this scenario, the maximum performance drop for FedscGen is 0.07. The second-best option would be 2 epochs for 10 rounds, which results in a maximum performance loss of 0.09 (Additional file 2: Fig. S1).

### FedscGen has a similar impact on downstream cell type classification as using scGen

After correcting the data using scGen, FedscGen, and FedscGen-SMPC models, we evaluated the effectiveness of batch effect correction in a downstream classification task. Specifically, we trained an MLP model to predict cell types from the latent representations of the corrected data using a cross-validation scheme, placing each batch in the test set once. The resulting classification accuracy differences show that FedscGen, with or without SMPC, performs equally well or better than scGen in the CL, MCA, and PBMC datasets. For the remaining datasets, the mean performance drop was not greater than 5%, demonstrating the utility of the federated secure solution compared to the centralized non-privacy-aware baseline (Fig. 2b).

### FedscGen performs comparably to scGen in different cell type inclusion scenarios

**Fig. 4:**
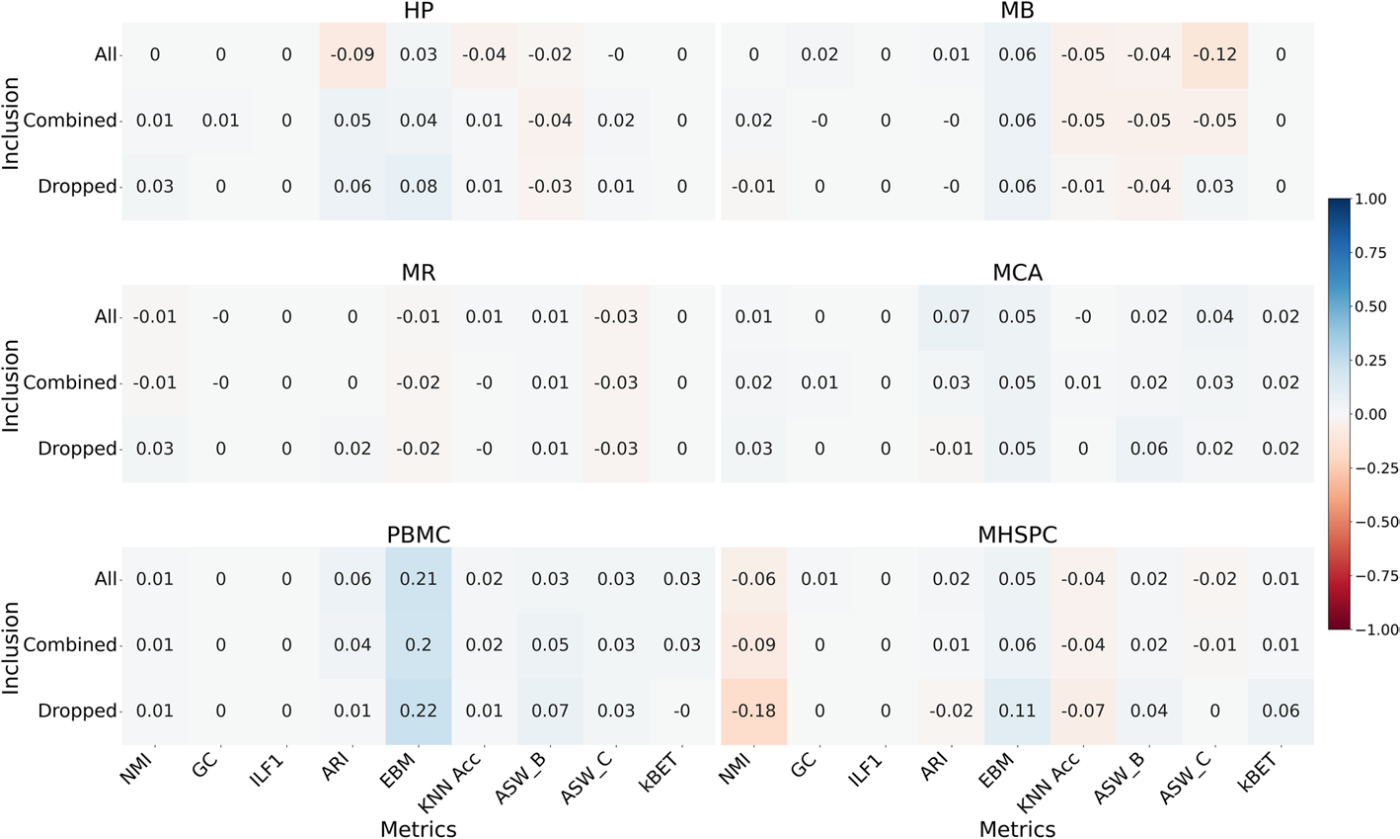
FedscGen yields comparable performance to scGen across *different cell type inclusion scenarios.* We evaluated performance differences between FedscGen and scGen under the batch-out-zero setting, where each batch in a dataset was treated as a client in federated workflows. Comparisons were made across three cell type inclusion scenarios—All, Combined, and Dropped—using six datasets: HP, MB, MR, MCA, PBMC, and MHSPC

Different studies, resulting in different batches, include various numbers of samples from specific cell types; some cell types are shared, while others appear only in one batch. Inclusion of standalone (only present in one batch) and minority cell types (with a few samples) in centralized batch effect correction experiments was previously done by combining [38] or using them as they appeared in the dataset [35]. Batch effect correction often relies on dominant batches per cell type, which raises the challenge of handling standalone cell types or, more broadly, the inclusion of minority cell types with few samples (Additional file 1: Tables S1–S2). To comprehensively evaluate FedscGen, we considered three different cell type inclusion scenarios: *All* (retaining all minority cell types), *Combined* (combining minority cell types and labeling them as “others”), and *Dropped* (removing minority cell types). We computed the performance difference $\triangle$ between FedscGen and scGen across nine metrics (NMI, GC, ILF1, ARI, EBM, KNN Acc, ASW_B, ASW_C, and kBET) over six datasets (Fig. 4 and Additional file 2: Fig. S3). In the MCA and PBMC datasets, FedscGen performs as well or better than scGen across all nine metrics, regardless of the inclusion scenario. For other datasets, FedscGen still shows non-negative performance differences across most metrics (Fig. 4). However, in the MB dataset for the KNN Acc and ASW_B metrics, and in the MHSPC dataset for the NMI and KNN Acc metrics, scGen outperforms FedscGen across all inclusion scenarios (Fig. 4). Notably, in the HP dataset for the ARI and KNN Acc metrics, switching from the *All* scenario to either the Dropped or Combined scenario leads to better FedscGen's performance (Fig. 4). Similarly, for the ASW_C metric in both the MB and MHSPC datasets, FedscGen performs as well as or better than scGen in the Dropped scenario, unlike in the All and Combined scenarios (Fig. 4). We also compared iLISI and cLISI across the three scenarios, observing highly similar results in both the Dropped and Combined scenarios (Additional file 2: Fig. S4). Additionally, we visualized the corrected data with UMAP for both scGen and FedscGen, compared with the raw data, for the Combined (Additional file 1: Fig. S5) and Dropped (Additional file 2: Fig. S6) scenarios.

## Discussion

In this study, we introduced FedscGen as a federated framework built on the scGen model to train a VAE in a privacy-preserving federated manner utilizing additive secret sharing for secure aggregation. Post-training, FedscGen runs a correction workflow to utilize the trained VAE model for correcting clients’ local data. In a comprehensive evaluation, we demonstrated that FedscGen is able to produce competitive results to its counterpart in a heterogeneous environment on eight different datasets. We applied multiple benchmarking criteria to point out any difference in terms of performance comparison of FedscGen and scGen; both models consistently deliver similar results in terms of batch mixing and cell type distinction.

### Benchmarking and performance trade-offs

Batch effect correction serves as a foundational step for a wide range of downstream analyses including clustering, classification, and differential expression, by harmonizing technical variability and enhancing comparability across datasets. In our comprehensive benchmarking, we used various metrics to evaluate the results of batch effect correction in scRNA-seq data, which can be broadly categorized into batch mixing/integration metrics (e.g., kBET, KNN-Acc, ASW_B, NMI, GC, and EBM), which assess how well cells from different batches are integrated, and biological signal preservation metrics (e.g., LISI, ASW_C, ARI, and ILF1), which evaluate the retention of true biological structure such as cell type identity [54]. It has been demonstrated that improving batch mixing can come at the cost of blurring biological distinctions, and vice versa [35, 54]. Accordingly, batch effect correction inherently involves a metric-driven trade-off: methods that aggressively remove batch effects may inadvertently eliminate biologically meaningful variation, while methods that preserve all biological signals might retain some batch-specific artifacts. Which method is preferable thus depends on which subset of evaluation metrics a practitioner chooses to prioritize—whether emphasizing integration or biological fidelity.

This observation aligns with findings from Tran et al. [35], who benchmarked 14 centralized batch correction methods—including scGen—and showed that no single method consistently outperformed others across all datasets and evaluation metrics. Accordingly, the performance of FedscGen, as a federated extension of scGen, should be interpreted in the context of potential performance trade-offs that may also arise when adapting other centralized methods to a federated setting. While the current study focuses on FedscGen, a natural next step would be to evaluate federated adaptations of other top-performing centralized methods using similar benchmarking criteria. Such comparative evaluation—guided by prior centralized benchmarking results—could further contextualize the strengths and limitations of FedscGen and help identify the most suitable approaches for privacy-sensitive real-world applications.

FedscGen’s distributed architecture, paired with its federated aggregation strategy, mitigates overfitting to batch-specific features—particularly under heterogeneous data conditions. As a result, in light of this trade-off, FedscGen occasionally achieves better integration without compromising critical biological structure—as observed, for instance, in the EBM and ASW_B metrics (Fig. 2d–e)—demonstrating that high performance in federated settings does not necessarily require access to pooled raw data.

To better understand these metric-driven trade-offs, we also examined the role of training configuration. Even though training FedscGen for eight communication rounds and two epochs shows competitive performance to centralized scGen across most metrics and datasets (Fig. 2), FedscGen’s performance is influenced by the choice of communication rounds and local training epochs. For instance, in the HP dataset, scGen outperforms FedscGen by 0.09 in ARI (Fig. 2b). The best FedscGen’s performance according to ARI occurs with 7 epochs and 1 round, making it 0.05 better than scGen (Additional file 2: Fig. S1). However, this improvement compromises KNN Acc and ASW_C, making FedscGen’s performance at least 0.05 worse than scGen. On the other hand, with 5 epochs and 6 rounds, the performance difference across six tuning metrics for FedscGen falls within − 0.01 to 0.06, effectively reducing the performance difference for ARI to zero. Accordingly, we recommend using eight communication rounds and two local epochs as a general-purpose configuration. For use cases requiring optimization for specific evaluation metrics or datasets, we provide a summary of the best-performing configurations in Additional file 1: Table S3. We note that we did not investigate variations in batch size or learning rate, as acceptable results were obtained using default values while tuning epochs and rounds. Despite the recommended configuration for FedscGen and the provided metric-prioritized choices of epochs and communication rounds for the evaluated datasets, tuning hyperparameters in a fully decentralized manner remains an open challenge—particularly in real-world, privacy-sensitive settings involving new or unseen data.

To further assess FedscGen’s robustness, we evaluated the impact of commonly used strategies in using minority and standalone cell types to evaluate batch correction methods. Different aspects of including all cell types in the experiments were covered in Figs, 1, 2 and 3. We extensively covered Combined (Additional file 2: Fig. S5) and Dropped (Additional file 2: Fig. S6) cell type inclusion scenarios, for combining and removing minority cell types, respectively, to contrast the performance of our federated model against scGen. In both Dropped and Combined scenarios, FedscGen and scGen show similar results in terms of batch mixing and cell type distinguishability across all datasets in benchmarking metrics (Fig. 4) and UMAPs (Additional file 2: Figs. S5–6). In fact, the comparable results across the majority of datasets in all scenarios had limited exceptions that show minor differences. Such performance differences can also be addressed by hyperparameter tuning in both scGen and FedscGen models. These results suggest that such performance differences are not inherent to the federated setup, but instead reflect the effect of tunable parameters that can be optimized based on the relative priority of technical correction or biological signal preservation.

Data heterogeneity across clients further complicates the metric-driven trade-offs. In our simulations, each client held data from one batch, resulting in variability in sample size, cell type composition, and sequencing protocols (Additional file 1: Tables S1–2). While changes to the DNN architecture could affect overall capacity, we found that fine-tuning the hyperparameters can help compensate for heterogeneity-related minor performance differences (Additional file 2: Fig. S1). Most hyperparameters, like learning rate and batch size, are shared across both models, and an early stopping mechanism is used for scGen. However, changing the number of local epochs and federated aggregations notably affects the FedscGen results. We trained the scGen model for 100 epochs which fully leverages the learning capacity of the model. On the other hand, we trained the FedscGen model for 8 communication rounds with 2 local epochs. Increasing the number of local epochs can make the model prone to overfitting for extremely non-iid data [30]. Therefore, adjusting the local epochs according to the heterogeneity level can improve the performance, albeit at the cost of increasing the number of communications. The VAE model in both scGen and FedscGen uses BatchNormalization layers which struggle with data heterogeneity in federated settings. Addressing shortcomings of the BatchNormalization layer was not within the scope of this study which can potentially further improve FedscGen results [55].

These trade-offs in performance tuning become particularly evident when considering the communication cost. In more homogeneous scenarios, where data distributions and sequencing protocols are aligned, fewer communication rounds may suffice to achieve competitive performance. The communication efficiency of FedscGen can be enhanced in accordance with data homogeneity. For instance, in a relatively homogeneous setting such as the MR dataset—characterized by uniform sequencing platforms, fairly balanced sample sizes, and consistent cell type distributions (Additional file 1: Tables S1–2)—FedscGen achieves comparable performance across all metrics in just two communication rounds. Remarkably, it reaches its best performance in ARI, NMI, EBM, and ASW_B with only one communication round (Additional file 2: Fig. S6), highlighting its communication efficiency when data heterogeneity is low.

Regarding benchmarking fairness, FedscGen primarily simulates federated scenarios to ensure computational scalability. Due to minor discrepancies in federated dimensionality reduction for UMAP [56], we opted to use centralized UMAPs across all methods to avoid biases introduced by differing evaluation strategies. This approach ensures that visual and quantitative evaluations reflect methodological performance rather than artifacts of implementation. While centralized evaluation supports fairness in benchmarking, federated evaluation remains the only viable option in real-world privacy-aware deployments. To support practical applications, we published FedscGen as a FeatureCloud app, which provides secure, containerized execution with built-in SMPC support in the platform.

Beyond benchmarking methodology and fairness considerations, we also examined how data characteristics—particularly the presence of minority or rare cell types—affect correction performance. Although previous studies have not systematically explored how different cell type inclusion strategies affect batch effect correction results, we addressed this gap by analyzing FedscGen’s performance across multiple inclusion scenarios to better contextualize our benchmarking results. While all main experiments in this study were conducted using the All scenario (where all cell types, including rare and standalone ones, were retained), we also evaluated performance under the Combined and Dropped scenarios to assess the method's robustness. Notably, in several datasets, FedscGen demonstrated improved or comparable performance to scGen when rare cell types were either merged or removed—particularly for metrics such as ARI and KNN Acc in the HP dataset (Fig. 4). This suggests that the All scenario represents the most challenging setup for FedscGen, and the observed performance gains in the Combined and Dropped scenarios underscore the strength of its competitive results even under the most complex inclusion strategy. Importantly, these findings highlight FedscGen’s adaptability and underscore the need for further systematic investigations into cell type inclusion strategies in future benchmarking studies.

Complementing this analysis, we further evaluated FedscGen’s utility in dynamic, real-world scenarios where new datasets may become available after initial model training. To assess FedscGen’s ability to integrate new studies in a privacy-aware manner, we evaluated its performance in a held-out batch scenario, where one batch was excluded from the training workflow and introduced only during correction. This simulates real-world settings where new studies may be integrated post hoc without retraining the model. In this setup, FedscGen successfully updated the mean latent features of dominant batches for shared cell types, enabling effective batch effect correction (Fig. 1c). Across all held-out scenarios, FedscGen performed comparably to scGen, with only minor differences observed in specific metrics (Fig. 1b). Importantly, FedscGen maintained both batch mixing and cell type separation quality similar to scenarios where all batches were included during training, demonstrating its robustness and practical utility for integrating new studies while safeguarding sensitive patient data (Fig. 1b–d).

To further examine the performance differences between FedscGen-SMPC, as a privacy-preserving method, and scGen, we conducted a systematic statistical analysis across seven datasets and eight metrics using the Wilcoxon–Mann–Whitney test. FedscGen-SMPC performed comparably to scGen, with no statistically significant differences observed across all datasets for GC and ILF1, while it significantly outperformed scGen in EBM and ASW_B across multiple datasets (Fig. 2e). In contrast, scGen significantly outperformed FedscGen-SMPC in certain metric–dataset combinations—for example, in ASW_C and ARI on the CL and MHSPC datasets—indicating better preservation of biological signals in these cases. Similarly, scGen showed significantly better performance in NMI on the HDC and MHSPC datasets (Fig. 2e). Despite these statistically significant differences, the observed performance gap remained within the range $\triangle_m\left[-0.07,\;0.19\right]$ across all tested metrics and datasets, highlighting the overall competitiveness of FedscGen-SMPC even in less favorable conditions and suggesting limited practical impact. Furthermore, the same trend of trade-offs observed earlier (Fig. 2d) is also apparent in this case of statistical testing (Fig. 2e), reflecting a balance between effective batch effect removal and the risk of inadvertently diminishing meaningful biological variation—an inherent challenge in batch correction.

### Privacy

Privacy concerns surrounding the sharing of scRNA-seq data arise not only from potential individual re-identification but also from broader institutional data governance challenges. While increased data availability significantly enhances the performance of machine learning models, especially deep learning, hospitals and research groups are often discouraged from data sharing due to the identifiable nature of expression profiles and the risk of revealing unpublished scientific findings. The privacy concerns addressed by FedscGen relate primarily to the protection of sensitive, potentially re-identifiable biological patterns in scRNA-seq data, rather than direct identifiers such as names or patient IDs. Even though single-cell count matrices may appear anonymized, emerging studies show that expression profiles can be used to infer phenotypes, identify rare or disease-specific cell types, or reconstruct genotype-associated signatures [21, 22]—raising the risk of indirect re-identification or the leakage of publishable insights [23, 24]. Beyond individual-level privacy, FedscGen addresses institutional restrictions rooted in scientific competitiveness, ethical obligations, and concerns over sharing unpublished results such as the discovery of novel or rare cell types, population-specific transcriptional profiles, or protocol-specific artifacts. While FedscGen does not share raw data or expose the learned representations, FedscGen-SMPC incorporates additive secret sharing, as an effective countermeasure [57, 58], to prevent model inversion or reconstruction attacks [59, 60].

In FedsGen, the coordinator role can be assigned to a trusted third party or to one of the participating clients. With SMPC in place, this coordinator never sees individual client updates, as they are masked and split across multiple computational parties. We used a default configuration of three computational parties, which can be increased to further minimize the risk of collusion. Despite the randomization introduced by secret sharing, which may cause minor variation across runs, FedscGen-SMPC achieves performance comparable to its non-encrypted counterpart across various datasets and metrics (Fig. 2b, c, e).

Once the training is complete, for correcting the local data, FedscGen offers two options: local update with precomputed mean latent features and federated correction, each with its computational and privacy implications. In the first option, by publishing mean latent features of the dominant batch for each cell type, the batch removal process can be done locally without revealing sensitive patients’ data since averaging masks the raw data. However, mean latent features are not updated with the inclusion of new studies with possible dominant batches for some cell types. Alternatively, FedscGen enables the participation of new studies in a correction workflow to effectively contribute dominant batches of new studies into the calculation of mean latent features. In fact, the federated correction workflow utilizes the trained model for removing batch effects, where the dominant batch for each cell type is identified using a privacy-preserving secret-sharing protocol across clients, and the corresponding mean latent features are shared for correcting local data with all clients (see the “ Methods” section). Accordingly, dominant batches are determined without revealing local cell-type counts; only the identity of the client holding the dominant batch for a given cell type is revealed to the aggregator. Although the model is not re-trained with new studies, we show that this strategy enables substantial correction using latent representations alone (Fig. 1d).

The privacy-preserving federated adaptation of alternative methods to scGen [18] requires careful consideration of both privacy and performance robustness, as the core mechanisms of statistical models may not directly translate to federated settings. For instance, federating Harmony [14]—a method that relies on iterative clustering and correction with centralized data access—necessitates the use of secure aggregation techniques such as secret sharing to maintain exact computations without leaking sensitive data. While secret sharing itself does not introduce noise, its application to iterative statistical procedures can pose challenges related to numerical precision, synchronization, and convergence in practice. This highlights the need for methodologically sound adaptations of statistical models that account for both privacy constraints and potential performance trade-offs in federated learning environments.

### Future directions

FedscGen’s performance in batch effect correction, while preserving patient privacy, underscores the potential of federated solutions, particularly with the advent of foundation models. This research can serve as a building block, potentially contributing to foundation models by enhancing feature extraction, nonlinear dimensionality reduction, and the integration of multi-modal data sources in a federated manner. Such advancements can enable a comprehensive understanding of cellular biology, spanning genomics, transcriptomics, epigenomics, and proteomics. Leveraging federated learning techniques, foundation models can be trained in a distributed manner on big datasets or fine-tuned with the inclusion of new studies, facilitating transfer learning and the adaptation of knowledge across domains to enhance scRNA-seq data analysis. Crucially, insights captured from integrated scRNA-seq data hold promise in constructing disease atlases, elucidating molecular mechanisms underlying diseases, and identifying potential therapeutic targets. Furthermore, integrated data serves as a valuable resource for supervised downstream tasks, including cell type classification, trajectory inference, and biomarker discovery, all of which can be performed in a federated fashion.

## Conclusions

In this study, we introduced FedscGen, a federated framework for batch effect correction in scRNA-seq data. FedscGen, built on the scGen architecture, employs additive secret sharing for secure aggregation of client models in a privacy-preserving manner. It avoids sharing raw patient data in compliance with GDPR and further protects learned parameters from leakage using SMPC. We demonstrated the effectiveness of FedscGen in removing batch effects through extensive benchmarking across eight diverse datasets and ten evaluation metrics, simulating heterogeneous scenarios by assigning different batches to different clients. FedscGen consistently achieved comparable performance to centralized methods, while metric-driven trade-offs through hyperparameter tuning enabled optimization under varying levels of data heterogeneity and communication constraints. By releasing FedscGen as a containerized FeatureCloud app, we facilitate its real-world adoption in multi-institutional research settings. This work lays the foundation for future privacy-preserving federated learning applications, particularly in federated workflows aimed at training large foundation models on harmonized, distributed single-cell data.

## Methods

FedscGen extends the scGen algorithm, which has demonstrated strong performance in batch effect correction for scRNA-seq data. Our framework enables collaborative batch correction by training local Variational Autoencoder (VAE) models across multiple clients in a federated learning setup. To ensure privacy, we employ additive secret sharing as a secure aggregation mechanism. FedscGen introduces two key workflows: a training workflow, where the VAE is collaboratively trained across clients, and a correction workflow, where mean latent features are used to compute a federated $\delta$-vectors shift for shared cell types across sites. We benchmark FedscGen against the centralized scGen model using diverse datasets and simulate heterogeneous conditions by assigning each batch to a separate client.

### scGen

The scGen algorithm (Additional file 3: Algorithm 1) takes as input the scRNA-seq data $\mathcal{X}$, which is a collection of samples from multiple batches $\mathcal{B}$. The scRNA-seq data undergoes normalization and scaling as a preprocessing step. A VAE model is initialized and subsequently trained using the training algorithm detailed in Additional file 3: Algorithm 2. The VAE comprises an encoder $\mathcal{E}$ that transforms the input data $x_i$ to a latent representation $z_i$, and a decoder $\mathcal{D}$ that reconstructs the data from the latent space. To approximate the posterior distribution $P\left(\left.x_i\right|z_i;\theta\right)$, with model parameters $\theta$, variational distribution $Q\left(z_i\left|x_i;\phi\right.\right)$ parameterized by $\phi$ is used. The training process involves optimizing the reconstruction loss $\mathcal{L}_{\text{recon}}$ and the Kullback–Leibler (KL) divergence loss $\mathcal{L}_{\mathrm{KL}}$ over a specified number of epochs and batch sizes.

$$\begin{aligned} &\mathcal{L}_{\text{recon}} (x_{i}) = \mathbb{E}_{z\sim Q (z| x_{i};\phi)} [\log P(x_{i}|z; \theta)] \\ &\mathcal{L}_{\text{KL}} (x_{i}) \leftarrow \text{KL} [{Q (z| x_{i};\phi)}\| P(z_{i}|x_{i};\theta)] \end{aligned}$$

Upon training completion, the trained VAE is used to correct batch effects using the centralized batch effect removal procedure, detailed in Additional file 3: Algorithm 3. After identifying shared cell types $\mathcal{T}$ and standalone cell types $\mathcal{S}$ (cell types that only appear in one batch), the average latent feature for each shared cell type $t$ is calculated as:

$$\mathcal M^t=\frac1{\left|Z_{b^\ast}^t\right|}{\textstyle\sum_{z\in Z_{b^\ast}^t}}z\;{\text{where}}\;b^\ast=argmax_{b\in B}N_b^t,\;\forall t\in\mathcal T$$

Next, shared cell types $t\in \mathcal{T}$ are corrected based on mean latent features $\mathcal{M}^t$ :

$$Z_{Corrected}^t\leftarrow Z^t-\mathcal{M}^t,\forall t\in \mathcal{T}$$

The correction procedure is described in the Additional file 3: Algorithm 4.

### FedscGen

We introduce FedscGen as a federated learning framework for scenarios where scRNA-seq data are distributed across different clients. In FedscGen, we ensure the raw data remains localized while we address privacy concerns. FedscGen as a cross-silo model supports federated collaboration among $\mathcal{C}$ clients with local scRNA-seq data $\mathcal{X}_c$ for client c and an initial model $\theta_{\mathrm{init}}$ with shared initial parameters across clients. Over $R$ communication rounds, each client trains a local VAE model for several epochs on their data. Each client communicates its model parameters $\theta_c$ and sample counts $\mathcal{N}_c$ to the coordinator for aggregation. The coordinator uses the $FedAvg$ aggregation method to update the global model $\mathrm{VAE}$ for the next round:

$$\theta_{r+1}\leftarrow{\textstyle\sum_{c\in C}}\left(\frac{N_c}{\sum_{c\in {\mathcal{C}}^{{\mathcal{N}}_c}}}\cdot\theta_c\right)$$

The updated model will be broadcast to all clients to resume their local training. This process is detailed in Additional file 3: Algorithm 5.

Following the federated training, a federated $\delta$-vector estimation and correction procedure is applied for correcting batch effects on local data without compromising privacy. Similar to the centralized scGen, the dominant batches $\mathcal{I}$ are identified by determining which client has the most number of cells for a given cell type, i.e., $\left|N_i^t\right|\geq\left|N_c^t\right|,\;\forall c\in\mathcal C\backslash\;i$. Accordingly, in the first aggregation step, clients $\mathcal{C}_\mathcal{I}^t$ with the dominant batch for cell type t will be detected and informed. Then, in the second aggregation step, each client will calculate and communicate the mean latent features of samples for any cell type which has dominancy:

$$\mathcal M^t=\mathrm{Avg}\left(\mathcal E\left(\mathcal X_{\mathcal C_{\mathcal I}^t}^t\right)\right),\forall\mathrm t\in\mathcal T$$

These mean vectors $\mathcal{M}$ act as federated $\delta$-vectors, representing the latent shifts used for correcting data across clients. Conditioned on the availability of $\mathcal{M}$ for all shared cell types at the client’s side, each client will be able to correct the batch effect on local data accordingly. For the standalone cell types $\mathcal{S}$, no mean latent features are calculated and therefore correction is not applied. The entire process is described in more detail alongside a comprehensive list of symbols and their descriptions in Additional file 3: Algorithm 6. If $\mathcal{M}^t$ and the scGen model is publicly available while the dominant batches are not affected by the arrival of new batches, the batch effect correction method can be applied in a centralized fashion.

To enhance privacy during model training and latent shift estimation, FedscGen employs secure multiparty computation (SMPC) using the CrypTen [61] framework. SMPC enables clients to jointly compute aggregated model parameters and latent statistics without revealing their locally trained models. For reproducibility during development and benchmarking, CrypTen was executed in debug mode using fixed random seeds and three computational parties for secure aggregation. We used FedAvg in FedscGen-SMPC to aggregate client models, weighting them by their sample sizes. To determine dominant batches for each shared cell type without exposing local cell-type counts, we implemented a secure SMPC protocol that computes the maximum across clients using secret sharing (Additional file 3). All experiments for FedscGen-SMPC were conducted using the same set of hyperparameters as FedscGen, for example, two local epochs and eight communication rounds.

### Preprocessing

Before performing batch effect correction, we filtered cells with very low coverage, fewer than 200 expressed genes, which are likely to correspond to empty droplets. Then, we normalized the data to have the same total counts per cell and selected highly variable genes, which are chosen based on their dispersion across the dataset. Only genes with a mean expression between 0.0125 and 2.5, and a dispersion greater than 0.7 are kept [35]. And finally, log1p transformation is applied to stabilize the variance across genes with a cap at a maximum value of 10. A summary of datasets for all scenarios is in Additional file 1: Table S1.

### Datasets

We analyze various datasets to compare FedscGen against scGen (centralized) in terms of batch correction. For the sake of simplification, we refer to the dataset by abbreviations. The Cell Line (CL) [39] dataset is derived from the 293t_jurkat experiment and contains three batches with 16,602 genes. Human Dendritic Cells (HDC) dataset comes from [40] and involves scRNA-seq data of human dendritic cells. Four cell populations were studied and two batches were created with slightly varying cell types, providing a challenge for batch correction. Human Pancreas (HP) dataset consolidated data from five sources [39, 41–45] with 14,767 cells each, and 15,558 genes. Mouse Brain (MB) [46, 47] dataset merges two mouse brain datasets with 691,600 and 141,606 cells, and 17,745 genes. Mouse Cell Atlas (MCA) dataset combines efforts from [48] and [49]. The focus is on 11 cell types from various organ systems. The batches consist of 4239 and 2715 cells, respectively, each containing 15,006 genes. Mouse Hematopoietic Stem and Progenitor Cells (MHSPC) dataset contains data from [50, 51] using the SMART-seq2 and MARS-seq protocols. Mouse Retina (MR) dataset combines the mouse retina data from two unassociated laboratories [52, 53] with 26,830 and 44,808 cells, and 12,333 genes. PBMC (human Peripheral Blood Mononuclear Cell) [39] dataset is based on scRNA-seq data of human PBMC with two batches that were made using the 3′ and 5′ 10 × Genomics protocols. The batches had 8098 and 7378 cells, respectively, each with 17,430 genes. For more detail see Additional file 1: Tables S1–2.

### Variational Autoencoder architecture

FedscGen builds upon the core architecture of scGen [18], which employs a Variational Autoencoder (VAE) combined with latent space arithmetic for effective batch effect correction. The VAE comprises a multilayer perceptron-based encoder and decoder, designed to project high-dimensional gene expression profiles into a lower-dimensional latent space while preserving meaningful biological variation. The encoder maps each input cell $\mathcal{X}\in R^d$ into a distribution over latent variables $\mathcal{Z}\in \mathcal{R}^k$, parameterized by a mean and variance learned through the network. The decoder then reconstructs the original expression vector from samples drawn from this latent distribution using the standard reparameterization trick. The loss function combines a reconstruction loss (mean squared error) with a Kullback–Leibler (KL) divergence term, regulated by a weighting factor $\alpha$ [62].

In our implementation, we adopt the same foundational design as scGen, using fully connected layers with ReLU activations. By default, the encoder and decoder each consist of two hidden layers of sizes 800 and 800, a latent dimensionality of 10, and a dropout rate of 0.05 applied to each layer. The decoder mirrors the encoder’s architecture in reverse. The number of input and output neurons corresponds to the number of highly variable genes selected from each dataset (Fig. 1a); for instance, for the CL dataset which contains 16,602 preprocessed genes, the input layer size will be 16,602 neurons. We used the same architecture consistently across both centralized and federated experiments for a given dataset, with fixed random seeds to ensure reproducibility.

### Experiments

As part of the comprehensive benchmarking of FedscGen against scGen, we designed experiments to investigate different influencing factors, including data heterogeneity and the inclusion of minority cell types. To simulate real-world data heterogeneity, we defined federated scenarios where each client had access to only one batch within a given dataset. Accordingly, each scenario includes a different number of clients depending on the number of batches. In general, we included all available batches in both training and correction workflows. However, to evaluate how well FedscGen can integrate new studies under real-world data heterogeneity conditions, we also conducted experiments using a held-out batch strategy. In this setup, the training workflow included all but one batch, with each participating batch assigned to a separate client. The remaining batch was excluded during training and introduced only in the correction workflow, alongside the other batches, to simulate the inclusion of a previously unseen study. This design enabled us to assess the robustness of FedscGen’s correction capabilities under data heterogeneity and its ability to generalize to new data without compromising correction quality.

To assess how the presence or absence of minority and standalone cell types affects batch effect correction, we implemented three distinct cell type inclusion scenarios: All, where all cell types—including minority and standalone—are retained as-is [35]; Combined, where minority and rare cell types are merged into a single label (“others”) [38]; and Dropped, where minority cell types are entirely excluded. These scenarios were applied across six datasets: HP, MB, MR, MCA, PBMC, and MHSPC. Following the approach in Lotfollahi et al. [38], we identified minority and standalone classes to determine eligibility for each scenario. Accordingly, the Combined and Dropped scenarios were not applicable to the HDC and CL datasets due to the absence of minority or standalone cell types. All experiments were conducted under a non-IID batch-out-zero federated setting, where each batch was assigned to a separate client in the training workflow. Notably, while batch sizes and distributions remain unchanged between the All and Combined scenarios, batch sizes may shrink in the Dropped scenario due to the exclusion of certain cell types.

### Downstream cell type classification

To evaluate the effectiveness of batch effect removal on downstream analysis using FedscGen (with or without SMPC) compared to scGen, we trained a multi-layer perceptron (MLP) classifier to predict cell types based on the latent representations produced by each method. The classifier architecture consisted of two hidden layers of size 800, each followed by batch normalization and ReLU activation, supporting stable training and improved generalization. The output layer was defined to match the number of unique cell types in the “all” inclusion scenario (Additional file 1: Table S2). All latent features were normalized using z-score normalization prior to being used for classification. The MLP models were initialized with a fixed seed for reproducibility and were trained using a cross-entropy loss function and optimized with the Adam optimizer. Meanwhile, we employed a leave-one-batch-out cross-validation strategy, where each batch was iteratively left out as a test set while the remaining batches were used for training. This experiment was conducted using FedscGen and scGen models that were first trained on the full set of batches and then used to correct the data before classification.

### Evaluation

To evaluate the effectiveness of batch effect correction, we employed a comprehensive set of metrics widely used in single-cell RNA-seq benchmarking studies. These metrics fall into two broad categories: those that assess batch mixing and those that evaluate the preservation of biological signal. Batch mixing metrics such as kBET, KNN accuracy (KNN-Acc), Average Silhouette Width for batch labels (ASW_B), Normalized Mutual Information (NMI), Graph Connectivity (GC), and Empirical Batch Mixing score (EBM) quantify how well cells from different batches are integrated following correction. Conversely, biological signal preservation metrics—including Local Inverse Simpson’s Index (LISI), ASW for cell types (ASW_C), Adjusted Rand Index (ARI), and Inverse Local *F*1 Score (ILF1)—measure the degree to which meaningful biological variation, such as cell type identity, is retained.

To quantify performance differences, we compared the performance of FedscGen against the centralized scGen baseline by computing the difference in each metric, defined as $\triangle{}_m={\mathrm{FedscGen}}_m-{\mathrm{scGen}}_m$. All calculated metric values throughout this study, lie in $m\in\left[0,1\right]$ resulting in $\triangle_m\in\left[-1,1\right]$. This provides an interpretable range where positive values indicate an advantage for FedscGen, and negative values indicate superior performance by scGen. The same strategy was used to assess FedscGen-SMPC relative to scGen. Performance difference was used throughout this study except for cLISI and iLISI, where the scores were compared directly.

To further evaluate FedscGen’s utility in scenarios involving the integration of new datasets after initial training, we designed a held-out batch experiment using the HP dataset. In this setup, a global model was trained on four clients, each representing a unique batch. During the correction workflow, the previously excluded fifth batch was introduced and corrected alongside the original four. This allowed us to test whether FedscGen could effectively update the mean latent features for each cell type in a federated manner, without requiring retraining of the VAE model (Fig. 1b, d). The experiment demonstrated that FedscGen can support dynamic correction workflows across institutions, highlighting its flexibility and real-world applicability.

To provide a robust statistical basis for these comparisons, we assessed the significance of performance differences $\triangle$ between FedscGen-SMPC and scGen, we performed a Wilcoxon–Mann–Whitney (WMW) test for each metric–dataset pair. Using WMW, we tested whether the performance scores from 10 independent runs (seeds) of each model were drawn from the same distribution, as a difference in distributions would suggest the observed performance differences are statistically significant. The test was applied to eight metrics across seven datasets, resulting in 56 independent comparisons to ensure comprehensive evaluation. *P*-values were computed for each metric–dataset pair, and to account for multiple testing, we applied the Benjamini–Hochberg (BH) correction to control the false discovery rate (FDR). Metric–dataset pairs with adjusted *p*-values below 0.05 were considered statistically significant. This approach provides a robust statistical basis for evaluating whether FedscGen consistently matches or outperforms scGen across diverse benchmarking scenarios.

#### k-Nearest neighbor batch-effect test (kBET) acceptance rate

Following Tran et al. [35], to quantitatively assess local batch mixing following dimensionality reduction, we employed the *k*-nearest neighbor batch effect test (*kBET*) [63, 64]. This statistical test evaluates whether cells from different batches are well-integrated in the latent space by comparing the batch label distribution among the *k*-nearest neighbors of each cell to the global batch distribution. Significant deviations from the global distribution indicate residual batch effects and inadequate mixing. kBET computes a *rejection rate* based on Pearson’s *χ*^2^ test applied to local neighborhoods. For each cell, the null hypothesis is that its local batch label distribution matches the global batch distribution. A low rejection rate (i.e., a high *acceptance rate*) suggests good batch mixing, while a high rejection rate indicates poor integration. The final *acceptance rate* is defined as the proportion of cells for which the null hypothesis is not rejected. We calculated kBET acceptance rates on the latent representations from FedscGen and scGen using the top 20 principal components (PCs) obtained from PCA. To ensure robustness, we followed the recommended strategy of computing acceptance rates across multiple *k*-values, representing different neighborhood sizes: 5%, 10%, 15%, 20%, and 25% of the dataset size (Additional file 2: Fig. S3). For each method and dataset, we report the median acceptance rate across these values (Figs. 1b and 2d). An exception was made for the Mouse Brain (MB) dataset due to its large size; here, *k* was fixed to 0.1% of the dataset size to maintain computational tractability.

#### Local Inverse Simpson’s Index (LISI)

To assess both batch mixing and biological signal preservation, in reduced-dimensional representations, we employed the Local Inverse Simpson’s Index (LISI), a metric designed to evaluate local neighborhood diversity in the latent space [63, 65]. LISI measures how well cells from different batches or cell types are integrated within their local neighborhoods based on nearest neighbors. This method first computes the local distance matrix and determines the k-nearest neighbors of each cell using a fixed perplexity value [63]. It then applies the inverse Simpson’s index, which captures the effective diversity of a label (e.g., batch or cell type) in a cell's local neighborhood. Higher iLISI values indicate greater diversity (i.e., effective batch integration). In contrast, lower cLISI values imply that cells of the same type cluster together (i.e., biological signal preservation). In our experiments, we calculated iLISI (integration LISI), which quantifies batch mixing where an iLISI score close to the number of batches suggests optimal batch integration. Furthermore, we calculated cLISI (cell type LISI), which quantifies the preservation of biological signals where a lower cLISI score (close to 1) indicates well-separated cell types in latent space, reflecting accurate biological clustering. We applied LISI to the top 20 PCs of raw and corrected data by each model. The iLISI score was computed only for shared cell types across all batches to ensure valid comparisons. The output consisted of one score per cell for each metric (Fig. 2c, Additional file 2: Fig. S4).

#### Average Silhouette Width (ASW)

To evaluate both batch effect correction and biological signal preservation, we applied the Average Silhouette Width (ASW), as implemented in prior work [38]. The silhouette score ranges from − 1 to 1 and quantifies how well a cell is embedded within its assigned cluster compared to neighboring clusters. Higher scores indicate that cells are well-clustered with similar cells and distinct from other clusters, suggesting strong cluster cohesion and separation. Using the top 20 PCs, we computed per-cell-type ASW (ASW_C), which uses cell type labels to evaluate the preservation of biological structure, and per-batch ASW (ASW_B), which uses batch labels to assess the extent of batch effect removal. A high ASW_C score reflects that cells of the same type form tight clusters, indicating successful preservation of true biological identity. In contrast, for ASW_B, lower silhouette scores are desired as well-corrected data should not cluster by batch. Therefore, we applied a normalization and reversal step to the ASW_B values so that higher normalized ASW_B scores correspond to better batch mixing. To account for batch effects within individual cell types, we further applied the cell-type-stratified ASW_B evaluation strategy [54, 66], where batch silhouette scores are computed within each cell type group and then averaged. This approach ensures that ASW_B reflects batch mixing independently of potential confounding by dominant cell types. Together, ASW_C and normalized ASW_B provide a balanced view of how well each method preserves biological signals while eliminating batch-driven artifacts in the latent space. Throughout our experiments we found $ASW\_C\in\left[0,1\right]$, and the same holds for ASW_B, enabling the computation of performance differences such as $\triangle{}_{\mathrm A\mathrm S\mathrm{W\_C}}={\mathrm{FedscGen}}_{\mathrm A\mathrm S\mathrm{W\_C}}-{\mathrm{scGen}}_{\mathrm A\mathrm S{\mathrm W}\_{\mathrm C}}$ which is consistent with the computation of performance differences for other evaluation metrics.

#### Adjusted Rand Index (ARI)

To quantify how well biological structure is preserved following batch correction, we used the Adjusted Rand Index (ARI) [38]. The ARI measures the similarity between two clusterings, adjusting for chance agreement. In our setup, we construct a KNN graph with *k* = 15 using the top 20 PCs, and then apply the Louvain algorithm [66] to infer clusters from the latent representation. These inferred clusters are compared to the known cell type labels using the ARI. A score of 1 indicates perfect concordance between the true and predicted clustering, while a score of 0 reflects random assignment. ARI is symmetric and remains invariant under label permutations, making it a robust metric for evaluating clustering alignment. It is particularly useful for assessing whether corrected data maintains cell type coherence while avoiding overclustering. Even though ARI score ranges from − 0.5 (indicating highly discordant clustering) to 1.0 (indicating perfect agreement), the observed ARI scores in our experiments consistently fell within $\left[0,1\right]$ range, requiring no additional normalization which facilitates direct comparison of ARI differences $\triangle{{}}_{ARI}$ with those derived from other evaluation metrics used in this study.

#### Entropy of Batch Mixing (EBM)

To evaluate local batch mixing in latent space, we employed the Entropy of Batch Mixing (EBM) metric [18, 54], which quantifies how uniformly cells from different batches are distributed among the nearest neighbors of a given cell. The EBM score is calculated by estimating the entropy of batch labels within local neighborhoods, thereby identifying residual batch-specific clustering. For each cell, we determined its 15 nearest neighbors using Euclidean distance and computed the Shannon entropy of the batch labels among those neighbors. This process was repeated across 100 randomly sampled cells, and the final EBM score was averaged across 50 independent sampling pools to ensure robustness. To allow comparability across datasets with different numbers of batches, the entropy values were normalized to lie within the interval $\left[0,1\right]$, where a score close to 1 indicates optimal batch mixing (i.e., high diversity among neighbors), and a score near 0 suggests strong batch-specific clustering and poor integration.

#### Normalized Mutual Information (NMI)

To evaluate the consistency between inferred and true cell type labels, we used the Normalized Mutual Information (NMI) metric [38]. NMI quantifies the mutual dependence between two clusterings, capturing how much information is shared between them. Specifically, we applied Louvain clustering using the top 20 principal components of the latent representation and compared the resulting clusters with known cell type annotations. To ensure fair comparison, we optimized the Louvain resolution to maximize the NMI score using the “arithmetic” averaging method [54, 66]. The final NMI value is calculated as the mutual information between the inferred clusters and the true labels, normalized by the average entropy of the two label distributions. A score of 1.0 indicates perfect agreement between clusters and labels, reflecting high biological fidelity, while a score of 0 indicates no mutual structure between the two partitions. NMI is symmetric and scale-invariant, making it suitable for comparing clustering outcomes across datasets of different sizes and complexities.

#### Isolated Label *F*1 (ILF1)

The Isolated Label F1 (ILF1) metric [54] is designed to assess how effectively rare cell type labels, which are least frequent across batches, are distinctly clustered in latent space. According to ScArches [48], we calculate the *F*1 score as the harmonic mean of precision and recall:

$$F_1=\frac{2\cdot\left(\mathrm{precision}-\mathrm{recall}\right)}{\mathrm{precision}+\mathrm{recall}}$$

where *F*1 ranges from 0 to 1 to contrast the isolated cell type against all others in the cluster. A higher score indicates pure clustering of the isolated label. When multiple isolated labels exist, their *F*1 scores are averaged to offer a comprehensive evaluation of the data's ability to capture nuanced biological distinctions. A higher ILF1 score (closer to 1) indicates that the isolated labels are well-separated from other cell types in the latent space, reflecting the model’s ability to preserve rare biological signals despite the presence of batch effects.

#### Graph Connectivity (GC)

We evaluate the structural coherence of cell types in the latent space using the Graph Connectivity (GC) metric [54]. For each cell type, we construct a *k*-nearest neighbor (kNN) graph based on the latent representation. We then compute the size of the largest connected component (LCC) in this graph and divide it by the total number of cells belonging to that cell type. This results in a per-type connectivity score ranging from 0 (completely fragmented) to 1 (fully connected). We considered the average GC score across all cell types, offering a unified measure of how consistently cells of the same type are clustered. Higher GC scores indicate more cohesive biological structure within cell types and improved preservation of biological identity following batch correction.

#### KNN accuracy

We evaluate KNN accuracy (KNN Acc) [38] as local purity of cell type clustering in the latent space, using top 20 PCs. For each cell, we identify its 15 nearest neighbors and compute the proportion that share the same cell type label, yielding an individual classification accuracy score. These scores are first averaged across all cells of each unique cell type, and then averaged across all cell types. A kNN Acc score closer to 1 indicates better local clustering of cells with the same label, reflecting a well-preserved biological structure in the latent space.

## Supplementary Information

Additional file 1: Supplementary tables for datasets and hyper-parameter choices

Additional file 2: Supplementary figures for benchmarking

Additional file 3: Supplementary methods

## Data Availability

All datasets used in this study were published in previously cited papers, and the preprocessed datasets are available for download at https://doi.org/10.5281/zenodo.11489844 [67] and the initial model weights used in our experiments are publicly available at https://doi.org/10.5281/zenodo.11505054 [68] for reproducibility. The source code for FedscGen is available at https://github.com/Mohammad-Bakhtiari/FedscGen, under the Apache 2.0 and BSD 3-Clause licenses. The reproducibility code is available at https://doi.org/10.5281/zenodo.15774785 [69]. The federated app is available in the FeatureCloud app store: https://featurecloud.ai/app/fedscgen.
