# Supplementary material for: FedscGen: privacy-preserving federated batch effect correction of single-cell RNA sequencing data
Source: Genome Biol. 2025 Jul 22;26:216. doi: 10.1186/s13059-025-03684-6 (PMC12285155; doi:10.1186/s13059-025-03684-6)
Supplement: Supplementary file 1 — Additional file 1: Supplementary tables for datasets and hyper-parameter choices [file 13059_2025_3684_MOESM1_ESM.pdf]

Datasets

In this study we used eight datasets for evaluating the performance of FedscGen and scGen models in terms of batch effect correction. The statistics of each datasets considering the preprocessing steps are give in Table:

Table S1: Dataset statistics

Dataset	Accession	N cells per batch	Technologies (1)	Source
CL (Cell Line)	293T	2,885	10X	http://scanorama.csail.mit.edu/data.tar.gz.
	Jurkat	3,258		
	50%:50% Jurkat:293T Cell Mixture	3,388		
HDC (Human Dendritic Cells) (2)	GSE80171	576	SMART-seq	https://www.ncbi.nlm.nih.gov/geo/query/acc.cgi?acc=GSE80171
HP(Human Pancreas) (1,3–7)	GSE85241	2,122	CelSeq2	https://hemberg-lab.github.io/scRNA.seq.datasets/human/pancreas/
	E-MTAB-5061	2,127	SMART-seq2	
	GSE84133	8,569	inDrops	
	GSE83139	457	SMARTer	
	GSE81608	1,492	SMARTer	
MB (Mouse Brain) (8,9)	GSE116470	691,600	Drop-seq	http://scanorama.csail.mit.edu/data.tar.gz.
	GSE110823	141,606	SPLiT-seq	
MCA (Mouse Cell Atlas) (10,11)	GSE108097	4,239	microwell-seq	https://www.ncbi.nlm.nih.gov/geo
	GSE109774	2,715	10X	
MHSPC (Mouse Haematopoietic Stem and Progenitor Cells) (12,13)	GSE81682	1,920	SMART-seq2	https://www.ncbi.nlm.nih.gov/geo

	GSE72857	2,729	MARS-seq	
MR(Mouse Retina) (14,15)	GSE81904	26,830	Drop-seq	https://hemberg-lab.github.io/scRNA.seq.datasets/mouse/retina/
	GSE63473	44,808		
PBMC(human Peripheral Blood Mononuclear Cell) (1)	3' dataset	8,098	10X	ftp://ngs.sanger.ac.uk/production/teichmann/BBKNN/PBMC.merged.h5ad
	5' dataset	7,378		

Table S2: Cell type inclusion statistics

Dataset	Cell type	Number of samples per cell	Standalone	Dropped/ Combined
HDC	CD1C	95	True	False
	CD141	94	True	False
	DoubleNeg	190	False	False
	pDC	190	False	False
MHSPC	CMP	548	False	False
	GMP	1148	False	False
	LMPP	280	True	True
	LTHSC	323	True	True
	MEP	1381	False	False
	MPP	368	True	True
	Unsorted	136	True	True
MCA	B-cell	1106	False	False

	Dendritic	220	False	True
	Endothelial	1134	False	False
	Epithelial	390	False	True
	Macrophage	469	False	False
	Monocyte	480	False	False
	NK	85	False	True
	Neutrophil	454	False	False
	Smooth-muscle	163	False	True
	Stromal	1032	False	False
	T-cell	1421	False	False
HP	MHC class II	5	True	True
	acinar	1368	False	False
	alpha	5100	False	False
	beta	3826	False	False
	delta	966	False	False
	ductal	1804	False	False
	endothelial	289	False	True
	epsilon	28	False	True
	gamma	656	False	False
	macrophage	55	True	True
	mast	32	False	True
	mesenchymal	107	False	True
	schwann	13	True	True
	stellate	511	False	True
	t_cell	7	True	True

PBMC	B cell	2371	False	False
	CD4 T cell	4450	False	False
	CD8 T cell	3142	False	False
	Hematopoietic stem cell	24	False	True
	Megakaryocyte	106	False	True
	Monocyte_CD14	4090	False	False
	Monocyte_FCGR3A	561	False	False
	NK cell	593	False	False
	Plasmacytoid dendritic cell	139	False	True
CL	293t	4490	False	False
	jurkat	5041	False	False
MR	amacrine	4668	False	False
	astrocytes	52	True	True
	bipolar	29766	False	False
	cones	1909	False	False
	fibroblasts	82	True	True
	ganglion	429	True	True
	horizontal	252	True	True
	microglia	67	True	True
	muller	4567	False	False
	pericytes	62	True	True
	rods	28534	False	False
	vascular_endothelium	250	True	True
MB	Astrocyte	67357	False	False
	Choroid_plexus	94	True	True

	Endothelial	46527	False	False
	Ependymal	2499	False	False
	Macrophage	1741	False	False
	Microglia	5717	False	False
	Mitotic	17	True	False
	Mural	12807	False	False
	Neurogenesis	2515	True	False
	Neuron	560672	False	False
	Olfactory ensheathing cells	178	True	True
	Oligodendrocyte	114719	False	False
	Polydendrocyte	17276	False	False
	Vascular and leptomeningeal cells	1087	True	False

Table S3: Optimal FedscGen Configurations by Dataset and Metric

Dataset	Metric	Best Round	Best Epoch	Performance Difference
**HDC**	ARI	6	2	-0.0017
	NMI	3	3	-0.0122
	EBM	1	1	0.3027
	ASW_B	2	7	0.0556
	ASW_C	2	1	0.0882
	KNN Acc	6	2	-0.0323
**MCA**	ARI	1	7	0.1369
	NMI	7	1	0.0376
	EBM	1	9	0.2531
	ASW_B	1	10	0.0218
	ASW_C	2	6	0.051

	KNN Acc	6	1	0.0012
**HP**	ARI	1	7	0.0456
	NMI	8	5	0.0214
	EBM	1	2	0.1987
	ASW_B	1	1	0.0326
	ASW_C	7	7	0.0275
	KNN Acc	8	5	-0.0242
**PBMC**	ARI	9	9	0.1847
	NMI	4	1	0.0463
	EBM	1	1	0.3442
	ASW_B	1	1	0.055
	ASW_C	2	1	0.0385
	KNN Acc	9	1	0.0251
**CL**	ARI	4	9	0.0691
	NMI	6	1	-0.0128
	EBM	1	10	0.1438
	ASW_B	1	1	0.0548
	ASW_C	3	1	-0.068
	KNN Acc	3	1	-0.0015
**MR**	ARI	1	8	0.1434
	NMI	1	2	0.0681
	EBM	1	10	0.0316
	ASW_B	1	9	0.0279
	ASW_C	2	1	-0.0197
	KNN Acc	9	2	0.0123
**MHSPC**	ARI	3	2	0.0762
	NMI	2	2	-0.0326
	EBM	1	5	0.1288
	ASW_B	2	2	0.0599
	ASW_C	4	6	-0.0015
	KNN Acc	4	1	-0.0258

References

1. Zheng GXY, Terry JM, Belgrader P, Ryvkin P, Bent ZW, Wilson R, et al. Massively parallel digital transcriptional profiling of single cells. Nat Commun. 2017 Jan 16;8(1):14049.

2. Villani AC, Satija R, Reynolds G, Sarkizova S, Shekhar K, Fletcher J, et al. Single-cell RNA-seq reveals new types of human blood dendritic cells, monocytes, and progenitors. Science. 2017 Apr 21;356(6335):eaah4573.

3. Baron M, Veres A, Wolock SL, Faust AL, Gaujoux R, Vetere A, et al. A Single-Cell Transcriptomic Map of the Human and Mouse Pancreas Reveals Inter- and Intra-cell Population Structure. Cell Syst. 2016 Oct;3(4):346-360.e4.

4. Muraro MJ, Dharmadhikari G, Grün D, Groen N, Dielen T, Jansen E, et al. A Single-Cell Transcriptome Atlas of the Human Pancreas. Cell Syst. 2016 Oct;3(4):385-394.e3.

5. Segerstolpe Å, Palasantza A, Eliasson P, Andersson EM, Andréasson AC, Sun X, et al. Single-Cell Transcriptome Profiling of Human Pancreatic Islets in Health and Type 2 Diabetes. Cell Metab. 2016 Oct;24(4):593–607.

6. Wang YJ, Schug J, Won KJ, Liu C, Naji A, Avrahami D, et al. Single-Cell Transcriptomics of the Human Endocrine Pancreas. Diabetes. 2016 Oct 1;65(10):3028–38.

7. Xin Y, Kim J, Okamoto H, Ni M, Wei Y, Adler C, et al. RNA Sequencing of Single Human Islet Cells Reveals Type 2 Diabetes Genes. Cell Metab. 2016 Oct;24(4):608–15.

8. Saunders A, Macosko EZ, Wysoker A, Goldman M, Krienen FM, De Rivera H, et al. Molecular Diversity and Specializations among the Cells of the Adult Mouse Brain. Cell. 2018 Aug;174(4):1015-1030.e16.

9. Rosenberg AB, Roco CM, Muscat RA, Kuchina A, Sample P, Yao Z, et al. Single-cell profiling of the developing mouse brain and spinal cord with split-pool barcoding. Science. 2018 Apr 13;360(6385):176–82.

10. Han X, Wang R, Zhou Y, Fei L, Sun H, Lai S, et al. Mapping the Mouse Cell Atlas by Microwell-Seq. Cell. 2018 Feb;172(5):1091-1107.e17.

11. The Tabula Muris Consortium, Overall coordination, Logistical coordination, Organ collection and processing, Library preparation and sequencing, Computational data analysis, et al. Single-cell transcriptomics of 20 mouse organs creates a Tabula Muris. Nature. 2018 Oct;562(7727):367–72.

12. Nestorowa S, Hamey FK, Pijuan Sala B, Diamanti E, Shepherd M, Laurenti E, et al. A single-cell resolution map of mouse hematopoietic stem and progenitor cell differentiation. Blood. 2016 Aug 25;128(8):e20–31.

13. Paul F, Arkin Y, Giladi A, Jaitin DA, Kenigsberg E, Keren-Shaul H, et al. Transcriptional Heterogeneity and Lineage Commitment in Myeloid Progenitors. Cell. 2015 Dec;163(7):1663–77.

14. Macosko EZ, Basu A, Satija R, Nemesh J, Shekhar K, Goldman M, et al. Highly Parallel Genome-wide Expression Profiling of Individual Cells Using Nanoliter Droplets. Cell. 2015 May;161(5):1202–14.

15. Shekhar K, Lapan SW, Whitney IE, Tran NM, Macosko EZ, Kowalczyk M, et al. Comprehensive Classification of Retinal Bipolar Neurons by Single-Cell Transcriptomics. Cell. 2016 Aug;166(5):1308-1323.e30.
