# Supplementary material for: FedscGen: privacy-preserving federated batch effect correction of single-cell RNA sequencing data
Source: Genome Biol. 2025 Jul 22;26:216. doi: 10.1186/s13059-025-03684-6 (PMC12285155; doi:10.1186/s13059-025-03684-6)
Supplement: Supplementary file 2 — Additional file 2: Supplementary figures for benchmarking [file 13059_2025_3684_MOESM2_ESM.pdf]

FedscGen Supplementary: Figures

1 HyperParameter tuning

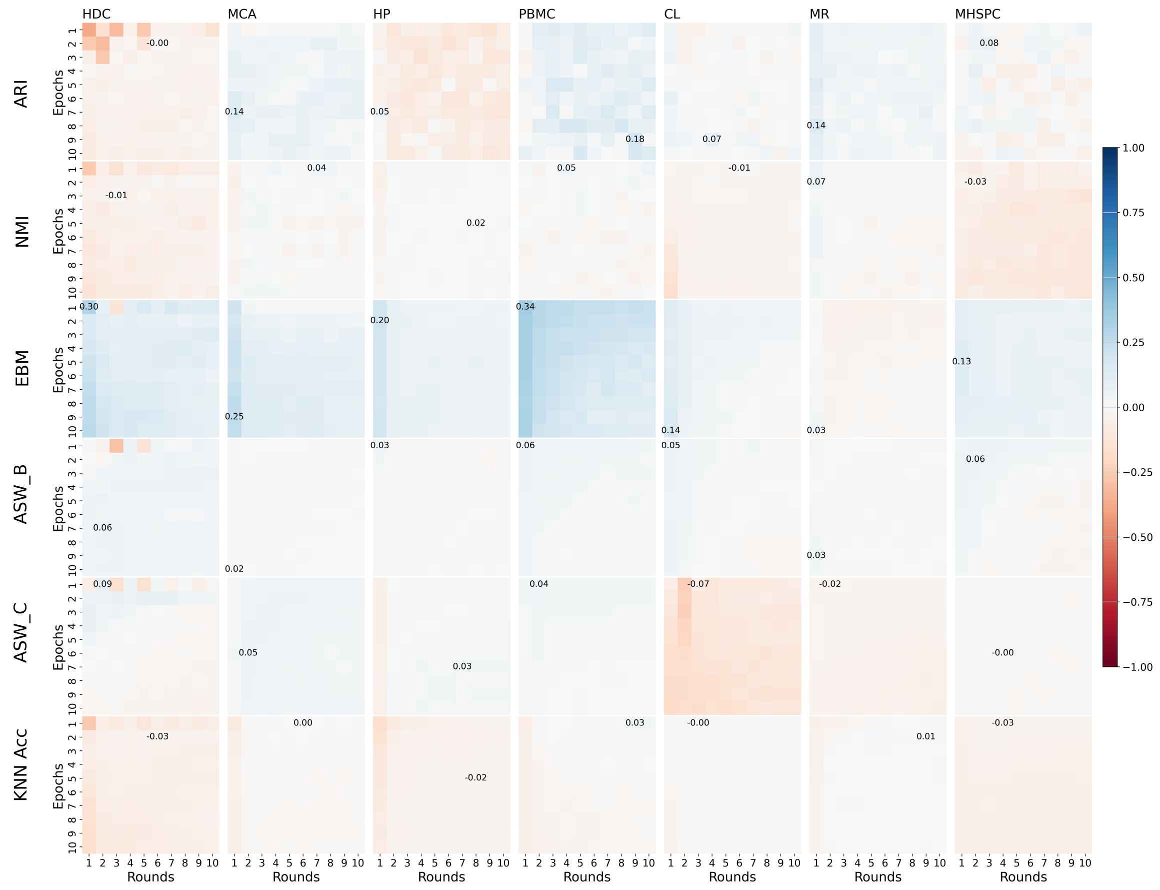

Fig S1: Tuning the number of communication rounds and local epochs significantly affects the performance of *FedscGen*. The model was trained from scratch across ten independent runs, evaluating performance after incrementally increasing local epochs from one to ten. During ten communication rounds, performance was benchmarked after global aggregation using multiple evaluation metrics.

Cell type inclusion scenarios

For some of the datasets, we have minority cell types, where some of them are *standalone cell types*, appearing only in one batch, that were dealt with differently in the community. For instance, *ScArches* [1] relabeled all the minority cell types as "other" before batch effect correction, and corrected them as if they all belong to the same cell type. On the other hand, some did not change cell type labels in the *Dataset* [2]. Since any

change in the unique cell types in a dataset can significantly affect the results, we evaluated the quality of batch effect removal with respect to considering all the reasonable strategies: *All*, *Combined*, and *Dropped*.

In this section, we compare all the approaches in three different settings to show that *FedscGen* is able to deliver comparable results to centralized batch effect correction using *scGen*. For each dataset, we extracted the list of minority samples. For *CL* and *HDC* datasets, there are no minority samples to combine or drop, so we present results only for the *All* setting.

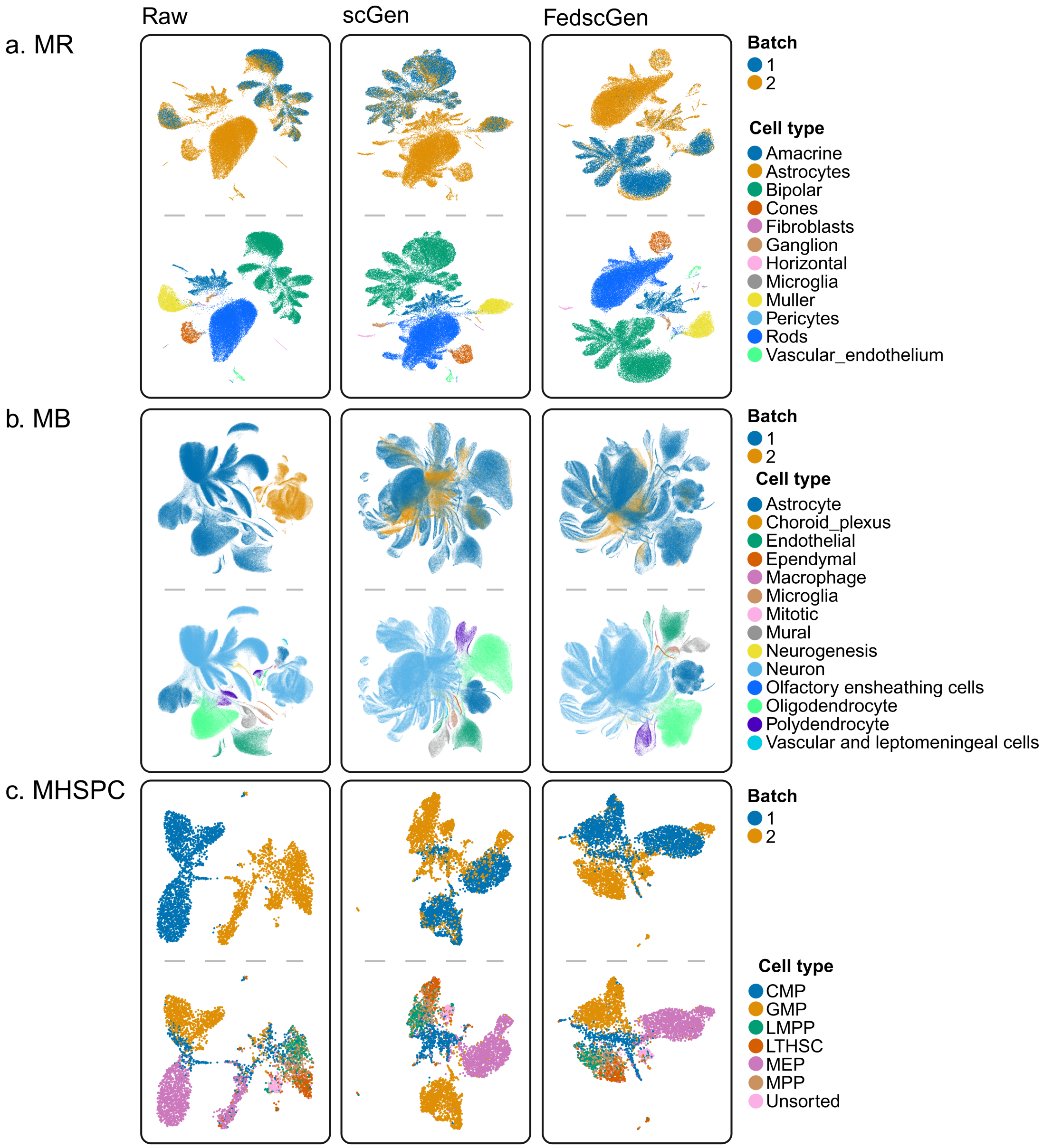

Fig S2: ***FedscGen* removes the batch effect by mixing batches and separating cell types very similar to *scGen*.** UMAPs of cell types and batches for raw and corrected data using *scGen* and *FedscGen* on **a)** *MR*, **b)** *MB*, and **c)** *MHSPC* datasets.

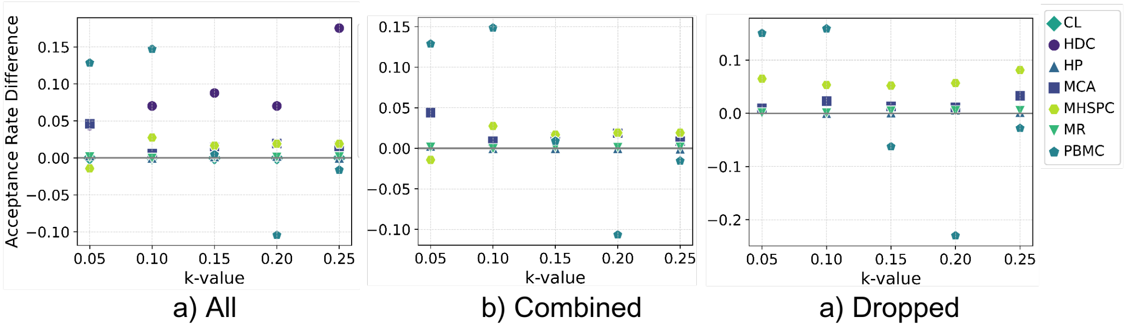

Fig S3: ***kBET acceptance rate difference* (acceptance rate of *scGen* minus acceptance rate of *FedscGen*)**. **a)** *All: FedscGen* outperforms *scGen* for *HDC* dataset across different k-values and with a smaller margin for the *PBMC* dataset at lower k-values, with no significant difference for the other datasets. **b)** *Combined: FedscGen* outperforms *scGen* for *PBMC* with a larger margin compared to the All setting, and also outperforms on *MCA*. **c)** *Dropped:* The margin of improvement decreases again for *PBMC*, while *FedscGen* outperforms *scGen* on *MHSPC*. For both Combined and Dropped settings, due to the absence of significant minority cell types in *CL* and *HDC*, those datasets are excluded.

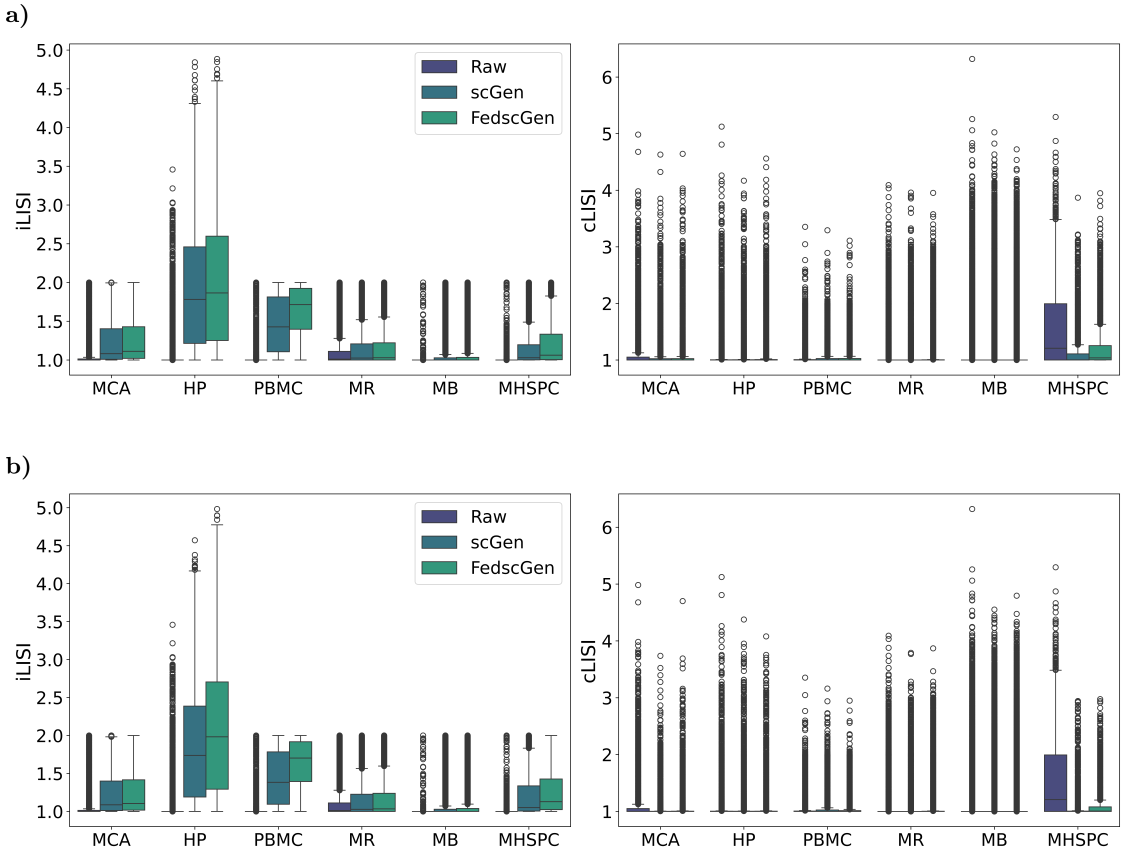

Fig S4: Comparison of different cell type settings for different datasets in **a)** Combined and **b)** Dropped conditions, evaluated using iLISI and cLISI metrics.

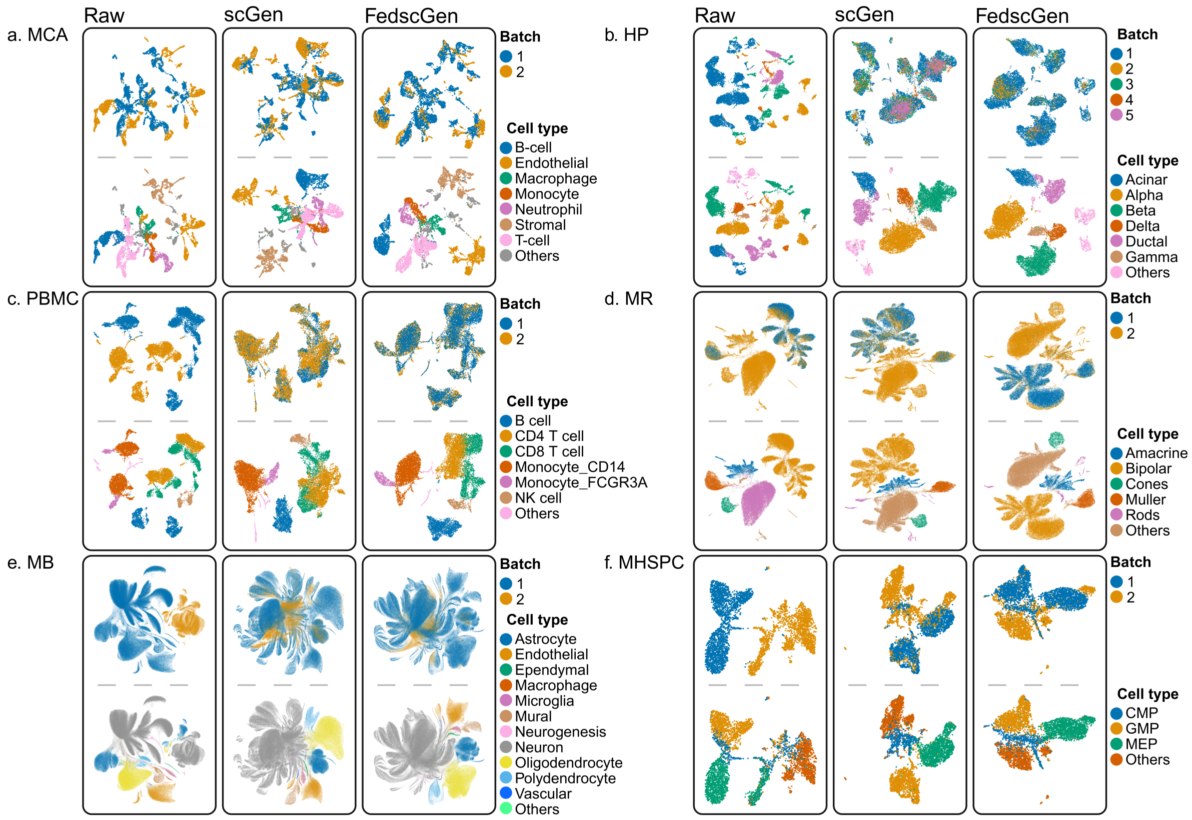

Fig S5: *FedscGen* removes batch effects as effectively as *scGen* in the *Combined* cell type inclusion scenario. **a)** *MCA*, **b)** *HP*, **c)** *PBMC*, **d)** *MR*, **e)** *MB*, and **f)** *MHSPC*.

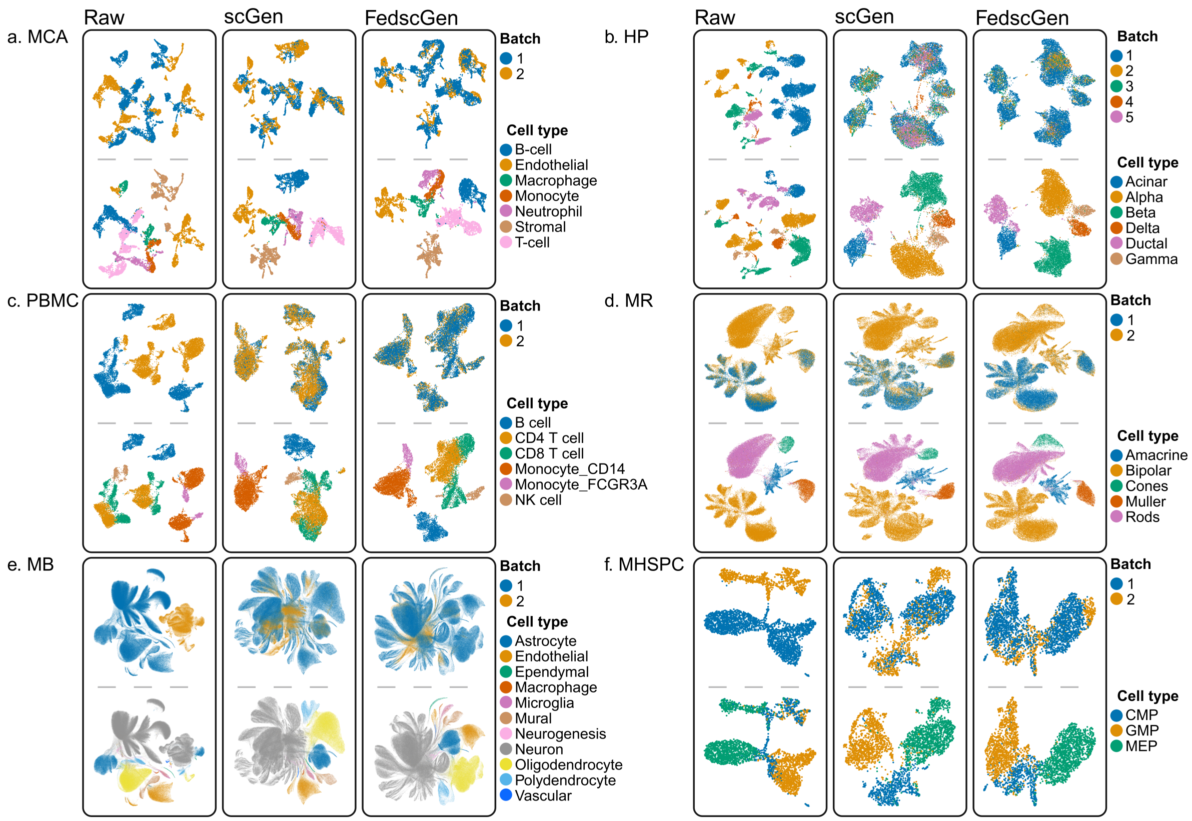

Fig S6: *FedscGen* removes batch effects as effectively as *scGen* in the *Dropped* cell type inclusion scenario. **a)** *MCA*, **b)** *HP*, **c)** *PBMC*, **d)** *MR*, **e)** *MB*, and **f)** *MHSPC*.

References

[1] Lotfollahi M, Naghipourfar M, Luecken MD, Khajavi M, Büttner M, Wagenstetter M, et al. Mapping single-cell data to reference atlases by transfer learning. Nat Biotechnol. 2022 Jan;40(1):121-30. Available from: https://www.nature.com/articles/s41587-021-01001-7.

[2] Tran HTN, Ang KS, Chevrier M, Zhang X, Lee NYS, Goh M, et al. A benchmark of batch-effect correction methods for single-cell RNA sequencing data. Genome Biol. 2020 Dec;21(1):12. Available from: https://genomebiology.biomedcentral.com/articles/10.1186/s13059-019-1850-9.
