# Supplementary material for: FedscGen: privacy-preserving federated batch effect correction of single-cell RNA sequencing data
Source: Genome Biol. 2025 Jul 22;26:216. doi: 10.1186/s13059-025-03684-6 (PMC12285155; doi:10.1186/s13059-025-03684-6)
Supplement: Supplementary file 3 — Additional file 3: Supplementary methods [file 13059_2025_3684_MOESM3_ESM.pdf]

FedscGen Supplementary: Method

ScGen Algorithm

The ScGen algorithm performs centralized batch effect correction for single-cell RNA sequencing (scRNA-seq) data using a variational autoencoder (VAE). It takes as input a set of gene expression matrices $\mathcal{X} = X_1, X_2, \ldots, X_b$, along with corresponding batch labels $\mathcal{B} = b_1, b_2, \ldots, b_n$ and cell type annotations T. The input data is first normalized and scaled to ensure comparability across cells. A VAE model is then trained using initial parameters θ_0, resulting in optimized parameters θ'. After training, batch effect correction is performed in the latent space via a centralized correction procedure. This step harmonizes the data across batches while aiming to preserve the underlying biological variation. The output is the batch-corrected gene expression matrix $\mathcal{X}_{\text{corrected}}$.

Algorithm 1 ScGen

```
Input:
𝒳 = {X_1, X_2, ..., X_b}: scRNA-seq data
ℬ = {b_1, b_2, ..., b_n}: Batch labels
T = {t_1, t_2, ..., t_n}: Cell types
θ_0 ← Initial parameters
Normalize and scale 𝒳
θ' ← TrainVAE(θ_0)
𝒳_corrected = CentralizedBatchCorrection( )
Output:
𝒳_corrected
```

TrainVAE Algorithm

The *TrainVAE* algorithm trains the Variational Autoencoder (VAE) used in both *scGen* and *FedscGen*. It initializes the encoder $\mathcal{E}$ and decoder $\mathcal{D}$ with model parameters θ, and iteratively updates them using stochastic optimization. For each mini-batch $\mathbf{x}_i \subset \mathcal{X}$, the encoder transforms the input into a latent representation $\mathcal{Z}$, and the decoder reconstructs it. The model is trained to minimize a loss consisting of the reconstruction loss $\mathcal{L}_{\text{recon}}$ and the KL divergence loss $\mathcal{L}_{\text{KL}}$, ensuring faithful reconstruction while regularizing the latent space. The algorithm returns the trained model parameters θ.

Algorithm 2 TrainVAE

```
Input:
θ ← Initial model parameters
Initialize encoder 𝓔 and decoder 𝓓 with θ
for i = 1 to epochs do
    for each batch x_i ⊂ 𝓧 do
        𝓩 ← 𝓔(x_i)                                   ▷ Encode to latent space
        x_i^recon ← 𝓓(𝓩)                              ▷ Reconstruct from latent
        𝓛_recon ← 𝔼_{z∼Q(z|x_i;φ)}[log P(x_i|z;θ)]
        𝓛_KL ← KL[Q(z|x_i;φ)‖P(z)]
        Update θ to minimize 𝓛_recon + 𝓛_KL
    end for
end for
Output: Trained model parameters θ
```

Centralized Batch Correction Algorithm

The *Centralized Batch Correction* algorithm is a core component of the *scGen* methodology, designed to address batch effects in *scRNA-seq* data using latent space transformations. Given a trained encoder $\mathcal{E}$, it first computes the latent representations $\mathcal{Z}$ of all cells. Then, it distinguishes between *standalone cell types*—those found in only one batch—and *shared cell types*, which appear across multiple batches.

For each shared cell type $t \in \mathcal{T}$, the algorithm identifies the *dominant batch*—the batch with the most cells of type t—and computes the mean latent representation $\mathcal{M}^t$ for that cell type in the dominant batch. These means are then used to shift the latent representations of corresponding cell types across other batches, thereby correcting for batch effects. The algorithm concludes by applying this correction to generate a batch-harmonized dataset.

Algorithm 3 Centralized Batch Correction

```
𝓩 ← 𝓔(𝓧)
I ← {t | count({b | 𝓝_b^t > 0}) = 1, ∀t ∈ T}          ▷ Standalone cell types
𝓣 ← T \ I                                           ▷ Cell types shared across batches
for each t in 𝓣 do
    𝓜^t ← Avg(𝓩^t_{arg max_b 𝓝_b^t})
end for
CorrectBatches(𝓜)                                   ▷ Batch effect correction
```

Correct Batches Algorithm

The *Correct Batches* algorithm is used in both the *scGen* and *FedscGen* pipelines. It takes, as input, the mean latent features $\mathcal{M}$ of shared cell types $\mathcal{T}$, and cor-

rects the latent representations $\mathcal{Z}$ accordingly. For *FedscGen*, the mean latent features are computed across clients in a federated manner.

The algorithm adjusts the latent representation for each shared cell type $t \in \mathcal{T}$ by subtracting the corresponding mean latent feature $\mathcal{M}^t$. The corrected latent representations are then combined with those of standalone cell types $\mathcal{I}$, which are left unchanged. Finally, the full corrected latent space is decoded using the decoder $\mathcal{D}$, producing the reconstructed, batch-corrected data.

Algorithm 4 Correct Batches

Input:
$\mathcal{M} \leftarrow$ Mean latent features
$\mathcal{Z}^t_{\text{corrected}} \leftarrow \mathcal{Z}^t - \mathcal{M}^t, \quad \forall t \in \mathcal{T}$ ▷ Correct shared cell types
$\mathcal{Z}' \leftarrow \bigcup_{t \in \mathcal{T}} \mathcal{Z}^t_{\text{corrected}} \cup \bigcup_{t \in \mathcal{I}} \mathcal{Z}^t$ ▷ Include standalone cell types
$\mathcal{X}_{\text{corrected}} \leftarrow \mathcal{D}(\mathcal{Z}')$ ▷ Reconstruct corrected data
Output: $\mathcal{X}_{\text{corrected}}$

FedscGen Algorithm

In the *FedscGen* algorithm each client $C_i \in \mathcal{C}$ retains its local dataset and collaboratively trains a shared *Variational Autoencoder (VAE)* model without exposing raw data. Training proceeds over $\mathcal{R}$ communication rounds. In each round, the current global model parameters θ_r are distributed to all clients. Each client trains the VAE locally using its own data and returns the updated model parameters and sample count $\mathcal{N}_i$ to the central coordinator. The coordinator aggregates these updates using a weighted average based on client sample sizes to produce new global parameters θ_{r+1}, which are redistributed for the next round. Once training is complete, a federated batch correction workflow is executed using the final trained model. This correction phase can incorporate new clients and applies the learned transformations to remove batch effects while preserving biological signal in a privacy-aware manner.

Algorithm 5 FedscGen

Input:
$\mathcal{C} = \{C_1, C_2, \ldots, C_m\}$: Set of clients
$\mathcal{R}$: Number of communication rounds
θ_1: Initial model parameters
for r = 1 to $\mathcal{R}$ **do**
    for each client $i \in \mathcal{C}$ **do**
        $\theta_i, \mathcal{N}_i \leftarrow \text{TrainVAE}(\theta_r)$
    end for
    $\theta_{r+1} \leftarrow \sum_{i \in \mathcal{C}} \left(\frac{\mathcal{N}_i}{\sum_{j \in \mathcal{C}} \mathcal{N}_j} \cdot \theta_i \right)$
end for
$\text{FederatedBatchCorrection}(\mathcal{C}, \theta_{\mathcal{R}})$

Federated Batch Correction Algorithm

The *Federated Batch Correction* algorithm extends the *FedscGen* framework to perform batch effect correction across a set of clients, which may include participants that were not involved in the training phase. The process begins by identifying the *dominant batch* for each shared cell type across all clients, based on the highest number of samples per cell type. Each client $C_i \in \mathcal{C}$ then computes the mean latent features $\mathcal{M}^t$ for cell types where it holds the dominant batch. These mean latent features serve as reference vectors for batch effect correction. Finally, each client applies the CorrectBatches algorithm using the aggregated mean latent features to remove batch-specific biases from their local latent representations. This process maintains the privacy of each client's raw data while enabling collaborative correction of batch effects.

Algorithm 6 Federated Batch Correction

```
Input:
C = {C_1, C_2, ..., C_m}: Set of clients
θ_trained ← Trained model parameters
Compute dominant batches for each shared cell type:
I = {arg max_{c∈C} N_c^t | t ∈ T}
Compute mean latent features for each dominant batch:
M^t = Avg(E(X^t_{I^t})),   ∀t ∈ T
for each client i ∈ C do
    CorrectBatches(M)
end for
```

Secure Determination of Dominant Batches Using SMPC

Dominant batches $\mathcal{I}$ are determined in a privacy-preserving manner under the SMPC mode using a secure aggregation protocol based on CrypTen, which relies on secret sharing to avoid leaking local cell-type counts. Each client $c \in \mathcal{C}$ locally counts its cell-type frequencies, resulting in a private vector of size $|T|$. These local count vectors are secret shared using CrypTen's SMPC backend, producing shared tensors $\tilde{x}_c$ for each client.

The secret-shared count vectors from all clients are stacked to form a matrix $\tilde{X} \in \mathbb{R}^{|\mathcal{C}| \times |T|}$, where each element $\tilde{X}_{c,t}$ represents the shared count of cell type $t \in T$ at client c. Then, for each t, the index of the maximum value is computed securely:

$$\mathcal{I}_t = \arg\max_{c \in \mathcal{C}} \tilde{X}_{c,t}$$

The result $\mathcal{I}_t$ indicates which client is dominant for cell type t, with all computation over cell counts performed entirely on secret shares, ensuring that no raw values are exposed to any party. For reproducibility, we handle ties with a

Table 1: Notations

Symbol	Description
$\mathcal{X}$	scRNA-seq data
$\mathcal{B}$	Batch labels
$\mathcal{X}_{\text{corrected}}$	Batch effect corrected data
$\mathcal{E}$	Encoder part of VAE
$\mathcal{D}$	Decoder part of VAE
$\mathcal{Z}$	Latent representation of data
$\mathcal{L}_{\text{recon}}$	Reconstruction loss
$\mathcal{L}_{\text{KL}}$	KL divergence loss
T	Set of unique cell types
t	Cell type
$\mathcal{S}$	Set of standalone cell types
$\mathcal{T}$	Shared cell types
$\mathcal{C}$	Set of clients
$\mathcal{R}$	Number of communication rounds
θ	Model parameters
$\mathcal{N}$	Number of samples
$\mathcal{I}$	Dominant batches
$\mathcal{M}$	Mean latent features

deterministic tie-breaking rule by assigning the cell type t to the lowest-indexed client without exposing additional information.
